# Tie2 activity in cancer associated myofibroblasts serves as novel target against reprogramming of cancer cells to embryonic-like cell state and associated poor prognosis in oral carcinoma patients

**DOI:** 10.1186/s13046-025-03405-8

**Published:** 2025-05-10

**Authors:** Paromita Mitra, Uday Saha, Kingsly Joshua Stephen, Priyanka Prasad, Subhashree Jena, Ankit Kumar Patel, Harshavardhan BV, Santosh Kumar Mondal, Sillarine Kurkalang, Sumitava Roy, Arnab Ghosh, Shantanu Saha Roy, Jayasri Das Sarma, Nidhan Kumar Biswas, Moulinath Acharya, Rajeev Sharan, Pattatheyil Arun, Mohit Kumar Jolly, Arindam Maitra, Sandeep Singh

**Affiliations:** 1 BRIC National Institute of Biomedical Genomics (BRIC-NIBMG), Kalyani, WB 741251 India; 2 Regional Centre for Biotechnology, Faridabad, India; 3 Indian Institute of Science Education and Research, Kolkata, India; 4 Indian Institute of Science, Bengaluru, India; 5 All India Institute of Medical Sciences, Kalyani, India; 6 Tata Medical Center, Kolkata, India; 7 Umea University, Umea, Sweden; 8 Comprehensive Cancer Center, University of Chicago Medicine, Chicago, IL USA

**Keywords:** Tumor microenvironment, Cancer associated fibroblasts, Tie2 signaling, TGFβ, Cancer stemness, Cancer plasticity

## Abstract

**Background:**

Myofibroblastic cancer-associated fibroblasts (CAF) in tumor stroma serves as an independent poor prognostic indicator, supporting higher stemness in oral cancer; however, the underlying biology is not fully comprehended. Here, we have explored the crucial role of Tunica Interna Endothelial Cell Kinase (Tie2/TEK) signaling in transition and maintenance of myofibroblastic phenotype of CAFs, and as possible link with the poor prognosis of head and neck squamous cell carcinoma (HNSCC) patients.

**Methods:**

Bulk and single cell RNA-sequencing (scRNAseq) methods and in-depth bioinformatic analysis were applied for CAF and cancer cells co-culture for studying molecular relationships. In vitro 3D-spheroid-forming ability, expression of stemness markers, in vivo tumor formation ability in zebrafish embryo and syngeneic mouse allografts formation was conducted to test stemness, upon targeting CAF-specific Tie2 activity by gene silencing or with small molecule inhibitor. Immunohistochemistry analysis was performed to locate the distribution of Tie2 and αSMA in primary tumors of oral carcinoma. Prognosis in HNSCC patient cohort from The Cancer Genome Atlas (TCGA) study was analysed based on single sample gene set enrichment score (ssGSEA) and Kaplan–Meier analysis.

**Results:**

Autocrine or exogenous TGFβ-induction in CAF led to the recruitment of histone deacetylase 2 (HDAC2) on the promoter of Tie2-antagonist, Angiopoietin-2 (ANGPT2), resulting in its downregulation, leading to phosphorylation of Tie2 (Y992) and subsequent activation of SRC (Y418). This led to SRC/ROCK mediated αSMA-positive stress-fiber formation with gain of myofibroblast phenotype. The CAF-specific Tie2-signaling was responsible for producing embryonic-like cell state in co-cultured cancer cells; with enhanced tumor initiating ability. Tie2 activity in CAF exerted the dynamic gene expression reprogramming, with the upregulation of ‘cell migration’ and downregulation of ‘protein biosynthesis’ related gene-regulatory-network modules in malignant cells. The AUCell scores calculated for gene signatures derived from these modules showed significant concordance in independently reported scRNAseq studies of HNSCC tumors and significant association with poor prognosis in HNSCC patient cohort.

**Conclusions:**

CAF-specific Tie2 activity may serve as direct stromal-target against cancer cell plasticity leading to poor prognosis of oral cancer patients. Overall, our work has provided wider applicability of Tie2-specific functions in tumor biology, along with its known role in endothelial cell-specific function.

**Supplementary Information:**

The online version contains supplementary material available at 10.1186/s13046-025-03405-8.

## Background

Cancer-associated fibroblasts (CAF) in tumor microenvironment (TME) are known to undergo changes that often promote tumor growth and survival [1]. In squamous cell carcinoma, stroma serves as a lifeline which provides essential nutrients, complex secretome of chemokines, cytokines and matrix forming (e.g. collagen) and degrading (e.g. matrix metalloproteinase) factors for tumor growth [2], and plays pivotal role in tumor metastasis [3]. Thus, modulation of activity of CAF to perturb their interactions with cancer cells have garnered attention and promised to innovate therapeutic strategies [4]; however, targeted therapy against CAF has remained to be elucidated. This may be primarily because CAF are heterogeneous population which may play context dependent roles [5, 6]. Though CAF are found to facilitate cancer progression, indiscriminate depletion of CAF has also shown to promote tumor growth [7]. Such paradoxical observation of CAF-functions warrants deeper understanding about its biology [8, 9].

Several lines of evidence have supported the notion that TGFβ produced in tumor microenvironment modulates adjacent fibroblasts into myofibroblasts [10–12] indicated by an increased expression of alpha smooth muscle actin (αSMA) and stress fiber formation [13, 14]. We have previously reported two diverse subtypes of CAF; C1-CAF (with lower score) and C2-CAF (with higher score) of αSMA-stress fiber-positive myofibroblasts in oral tumors, where C2-CAF supported higher stemness in cancer cells [15]. Stemness is defined as the ability of cancer cells to display long-term regeneration ability, giving rise to heterogeneous subpopulations of cancer cells; linked with cancer initiation, progression and poor treatment responses [16–18]. Targeting these stem-like cancer cells (SLCCs) may be crucial for overall success of treatments [19, 20].

Tunica interna endothelial cell kinase 2 (Tie2) gene, also known as TEK or angiopoietin-1 receptor, encodes for a receptor tyrosine kinase. Substantial reports have suggested ANGPT1 as agonist of Tie2 signalling [21, 22]. However, ANGPT2 acts as antagonist at its higher concentration or may also act as agonist in the absence of ANGPT1 [23–25]. Studies on Tie2 pathway have been majorly focused on endothelial cell functions, related to vessel maturation and vascular integrity [26, 27]. Increasing literature have gathered evidences of Tie2-activation in pericytes, macrophages and hematopoietic stem cells, as well [28–30]. Role of Tie2 in cancer tissue is reported in breast tumor-bone microenvironment, where Tie2-positive myeloid cells were found to be involved in osteoclast differentiation and osteolytic bone invasion of murine breast cancer cell line [31]. Also, elevated ANGPT1/Tie2 signaling was positively correlated with increased cell proliferation and migration in thyroid carcinoma [32]. Tie2-positive cervical cancer cells are recently reported to induce VEGFR2 and Tie2 expression in endothelial cells and can promote angiogenesis [33]. Moreover, Tie2-expressing cervical cancer cell-derived exosomes transport Tie2 protein to infiltered macrophages, and thereby increase angiogenesis [34]. Similarly, neovascular endothelial cells showed higher expression of Tie2 in hepatocellular carcinoma [35]. Tie2 expression in oral tumor tissues is studied briefly [36]. Additionally, Tie2 was also among the top upregulated genes in patient derived C2-CAF in our earlier report [15]; however, its fibroblasts specific expression and precise role in the biology of oral tumor microenvironment has remained to be elucidated.

Here, we report that Tie2 activity was found essential for the initiation and maintenance of TGFβ-induced myofibroblastic differentiation and acquisition of the transcriptional state of C2-CAF. Furthermore, Tie2-signal in C2-CAF was responsible for reprogramming oral cancer cells to acquire embryonal gene expression state with increased stemness and epithelial to mesenchymal transition (EMT) status. Validating our in vitro results, similar CAF-induced cancer cell reprogramming was also identified in HNSCC tumors at single cell level, and found associated with poor prognosis in TCGA-HNSCC patient cohort, suggesting the clinical implication of our study. Targeting Tie2-activity in oral-CAF led to reduced tumorigenic ability of cancer cells; demonstrating wider applicability of Tie2, beyond endothelial cell specific functions.

## Results

### C2-CAF expressed higher levels of Tie2 and positively correlated with αSMA-high stromal fibroblasts in primary tumors

Provided that TGFβ induces myofibroblastic differentiation, and based on our previous study where C2-CAF demonstrated myofibroblastic phenotype; we first tested if C1-CAF may acquire status of C2-CAF upon TGFβ induction. Interestingly, stimulation of TGFβ (10 ng/ml) led to a significant increase in frequency of cells having αSMA-positive stress fibers in all three tested, patient-derived C1-CAF; indicative of myofibroblastic differentiation (Figure S1 A). Moreover, TGFβ-induction resulted in gain of C2-CAF associated genes (*FN1, SERPINE1, ITGB1*); while, genes associated with C1-CAF state (*FOXF1, EYA1, RUNX2*) were downregulated compared to untreated control, suggesting TGFβ-induced transition of C1-CAF to C2-CAF status (Figure S1B). αSMA is associated with contractile apparatus of smooth muscle cells and myofibroblasts and exhibits matrix remodelling ability [34]. Notably, TGFβ-induced CAF had better matrix remodelling ability than untreated C1-CAF group (Figure S1 C, i-ii). Taken together, TGFβ-induction clearly converted C1-CAF (αSMA^low^) to C2-CAF (αSMA^high^). For ease of understanding, we have labelled C1-CAF as UT-CAF and TGFβ-induced C1-CAF as TGF-CAF.

**Fig. 1:**
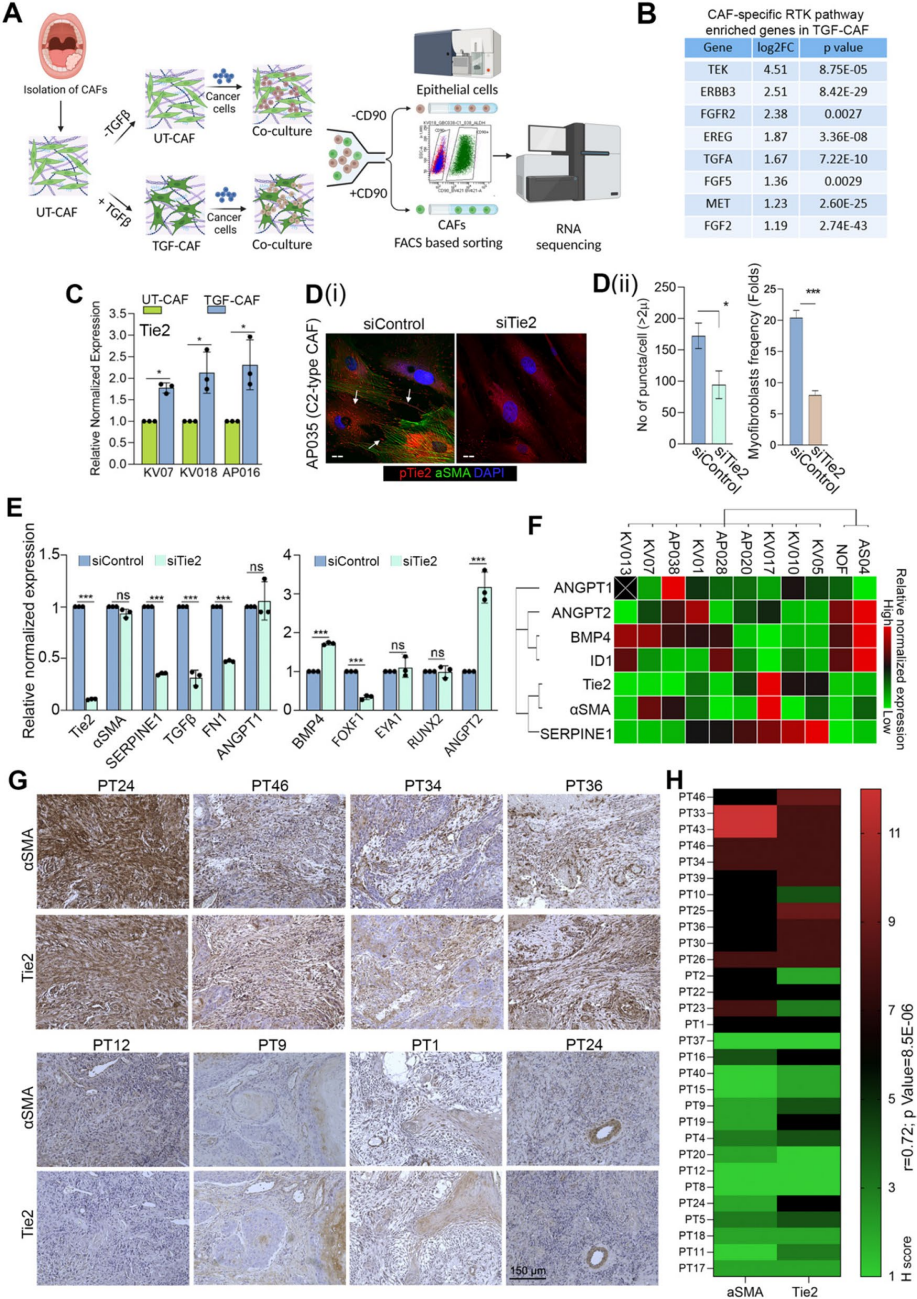
C2-CAF expressed higher levels of Tie2 and positively correlated with αSMA-high stromal fibroblasts in primary tumors.(**A**) A schematic depicting the experimental design for co-culture of UT-CAF and TGF-CAF with cancer cells and downstream processing. (**B**) List of eight common upregulated genes between RTK, PI3 K, MAPK in TGF-CAF. (**C**) qPCR analysis of *Tie2* in three different primary CAF under untreated (UT-CAF) or 10 ng/ml TGFβ-induced (TGF-CAF) conditions. (**D**) (i) Images of constitutively activated C2-CAF (AP035), stained for aSMA (green), pTie2 (Y992) (Red), and nucleus (DAPI, purple) after RNAi mediated silencing of Tie2 (siTie2). Scrambled siRNA (siControl) was used as a control. Arrowhead indicates pTie2 (Y992) positive puncta. (ii) frequency of CAF with myofibroblast-phenotype (with aSMA- positive stress fiber) and pTie2 (Y992) puncta was quantified using ImageJ. Scale bars, 20 µm. (**E**) qPCR analysis of C1-CAF related genes (*BMP4, EYA1, RUNX2, FOXF1, ANGPT2*) and C2-CAF related genes (*Tie2, TGFꞵ, SERPINE1, *a*SMA, FN1, ANGPT1*) in constitutively activated C2-CAF following Tie2 knock-down. (**F**) Heatmap showing qPCR-based expression of C1- and C2- CAF related genes across different primary CAF from oral cancer patients and normal oral fibroblasts. (**G**) Representative images of human oral tumor tissues detected for αSMA and Tie2 protein expression using IHC. (**H**) Heatmap showing correlation between H-score of αSMA and Tie2 protein in oral tumor stroma. Scale bars = 20 µm. *P<0.05, **P<0.01, ***P<0.001

To explore tumor-stroma interaction, UT-CAF or TGF-CAF were co-cultured with cancer cells. Following co-culture, cells were separated using FACS and bulk-RNAseq was performed on sorted cells, subsequently (Fig. 1A). We found that 886 and 1065 genes were upregulated and downregulated respectively (log2 FC > 1, *p* value ≤ 0.05) in TGF-CAF compared with UT-CAF (Table S1). Gene set enrichment [37], with Cytoscape analyses, suggested the enrichment of key regulatory pathways involving RTKs, PI3 K/AKT, focal adhesion, JAK-STAT pathway, cytokines- and interleukins-mediated pathways (Table S2) in TGF-CAF (Figure S2 A-D). Receptor tyrosine kinases (RTKs) are key regulatory trans-membrane receptors which made them suitable candidates for therapeutic target [38]. Activation of RTK leads to downstream activation of MAPK and PI3 K-AKT pathway. With the aim to identify the common regulators among RTKs; *TEK* (*Tie2)*, *ERBB3*, *FGFR2*, *EREG*, *TGFA*, *FGF5*, *MET*, and *FGF2* were commonly upregulated in TGF-CAF (Fig. 1B, S2E), with Tie2 being the most upregulated RTK among these common genes. Also, genes associated with Tie2 signaling were significantly enriched in TGF-CAF (Figure S2 F-i) and Tie2 upregulation was verified by qPCR (Figure S2 F-ii). This observations collectively prompted us to explore the expression and function of Tie2 in oral-CAF in response to TGFβ.

Interestingly, after TGFβ treatment, direct upregulation of Tie2 was observed in all tested CAF, independent of co-culture with cancer cells (Fig. 1C). Thus, we first explored the Tie2 association with myofibroblastic phenotype and maintenance of C2-like state of CAF. Interestingly, silencing of Tie2 in patient derived C2-CAF resulted in significant loss of myofibroblasts frequency, compared to control (Fig. 1D, i-ii). Reduced Tie2-phosphorylation (Y992) of the activation loop suggest the downregulation of Tie2-activity [39, 40]. Upon Tie2-silencing we observed the loss of phosphorylated Tie2 (Y992) puncta, possibly due to the reduced number of mature focal adhesions [41]. Importantly, Tie2 silencing in C2-CAF also resulted in concomitant downregulation of tested C2-CAF related genes (*SERPINE1, FN1, TGF*β) whereas the C1-CAF related gene *BMP4* was upregulated. Additionally, ANGPT2 was upregulated in Tie2 silenced C2-CAF without having any effect on its agonist, ANGPT1 (Fig. 1E). Therefore, to substantiate, we further explored this correlation in ten different oral tumor derived CAF and normal oral mucosal fibroblast (NOF). Interestingly, gene expression based unsupervised clustering grouped *Tie2* with *αSMA* and *SERPINE1*; whereas *ANGPT2* clustered with *BMP4* and its downstream gene *ID1*. *ANGPT1* expression did not specifically associate with any specific group (Fig. 1F). Collectively, results established a strong correlation between Tie2 expression with myofibroblastic C2-like state of CAF. Encouraged from these results, we evaluated the tumor stromal expression of αSMA and Tie2 on serial sections of surgically resected human oral tumor tissues (*n* = 30) (Fig. 1G). To our interest, we observed significantly higher H-score of Tie2 in tumors having αSMA-high stromal fibroblasts as compared to tissues having αSMA-low stroma (Fig. 1H); strongly suggesting the presence of Tie2-positive CAF in oral tumor stroma.

### Tie2 plays an essential role in induction as well as sustenance of TGFβ-induced myofibroblastic differentiation of CAF

**Fig. 2:**
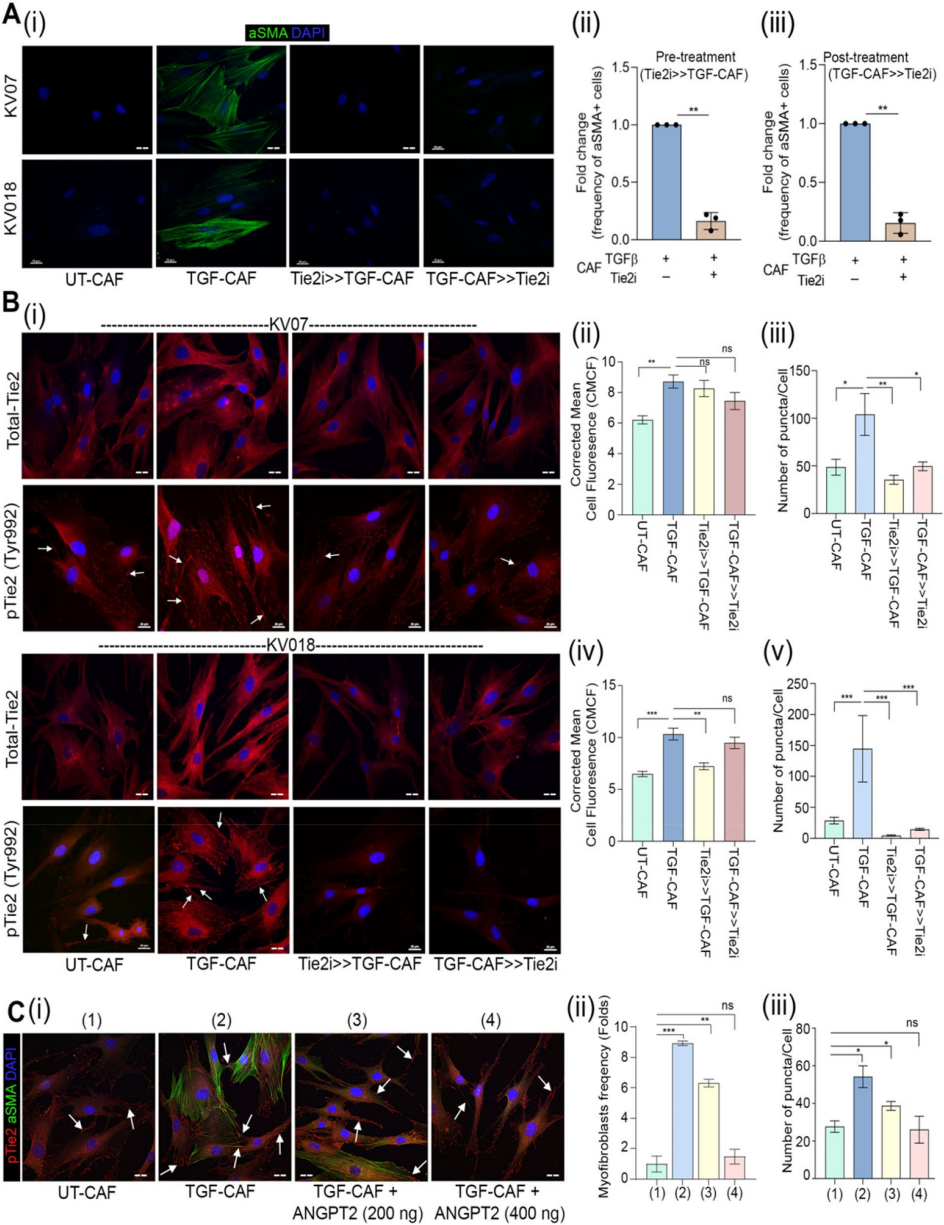
Tie2 plays essential role in induction as well as sustenance of TGFβ-induced myofibroblastic differentiation of CAF. (**A**) (i) Representative images and quantification of myofibroblasts frequency in UT-CAF and TGF-CAF. Tie2-inhibitor was added 1 h before TGFβ induction (Tie2i > > TGF-CAF) or 48 h after TGFβ induction (TGF-CAF > > Tie2i). Cells were quantified using ImageJ. (ii-iii) Frequency of αSMA stress fibre-positive cells were plotted for three different patient derived CAF. (**B**) (i) Representative images of Tie2 and pTie2 (Y992) in UT-CAF and TGF-CAF after Tie2-inhibition for 1 h before (Tie2i > > TGF-CAF) or 6 h after TGFβ induction (TGF-CAF > > Tie2i). (ii-v) Bar graph showing quantification of total Tie2 protein and pTie2 (Y992) puncta, calculated using ImageJ software. (**C**) (i) Representative images of αSMA and pTie2 (Y992) in UT-CAF, TGF-CAF or with increasing doses of ANGPT2 (200 ng/ml, 400 ng/ml) in the presence of TGFβ. Arrowhead indicates pTie2 (Y992) puncta. (ii) Bar graph showing cell frequency with αSMA stress fiber-positive CAF and (iii) pTie2 (Y992) expression by CAF in given conditions. Scale bar = 20 µm. *P < 0.05, **P < 0.01, ***P < 0.001

Tie2 silencing experiment clearly suggested that Tie2 function may be required for TGFβ-induced myofibroblastic differentiation. We next used a commercially available small molecule inhibitor, selective against Tie2 kinase (Tie2i) [42]. Similar to our observation with Tie2 silencing, one hour pre-treatment with Tie2i before TGFβ induction showed significantly less frequency of myofibroblasts compared to DMSO control (Fig. 2A) in two different patient-derived C1-CAF. More importantly; even after CAF were successfully induced to myofibroblasts by TGFβ, Tie2i effectively reversed this myofibroblast phenotype (Fig. 2A) and downregulated C2-CAF associated genes *αSMA, SERPINE1* and *Tie2* (Figure S3 A, S3B).

Further, upon TGFβ-induction a significant increase in total-Tie2 protein and frequency of phosphorylated-Tie2 (Y992) puncta (Figure S3 C (i, ii)) were observed for tested CAF (Fig. 2B, i-v). Importantly, one hour pre-treatment with Tie2i before TGFβ induction as well as six hour of Tie2-inhibition after complete myofibroblastic differentiation by TGFβ (post-treatment), both conditions showed reduced number of Tie2-phosphorylated puncta. Since, ANGPT2 is a known to act as an antagonist of Tie2-receptor activation, we next used soluble ANGPT2 to inhibit Tie2 signaling. Very interestingly, similar to Tie2i, reduced frequency of myofibroblasts (Fig. 2 C, i-ii) and number of Tie2-phoshorylated puncta (Fig. 2 C-iii) was observed after ANGPT2 addition. Taken together, results provided novel insights, where CAF-specific Tie2 activity was responsible for induction and maintenance of TGFβ-induced myofibroblastic phenotype as well as transition to transcriptional state of C2-CAF.

### Tie2-activity is regulated in an autocrine manner

**Fig. 3:**
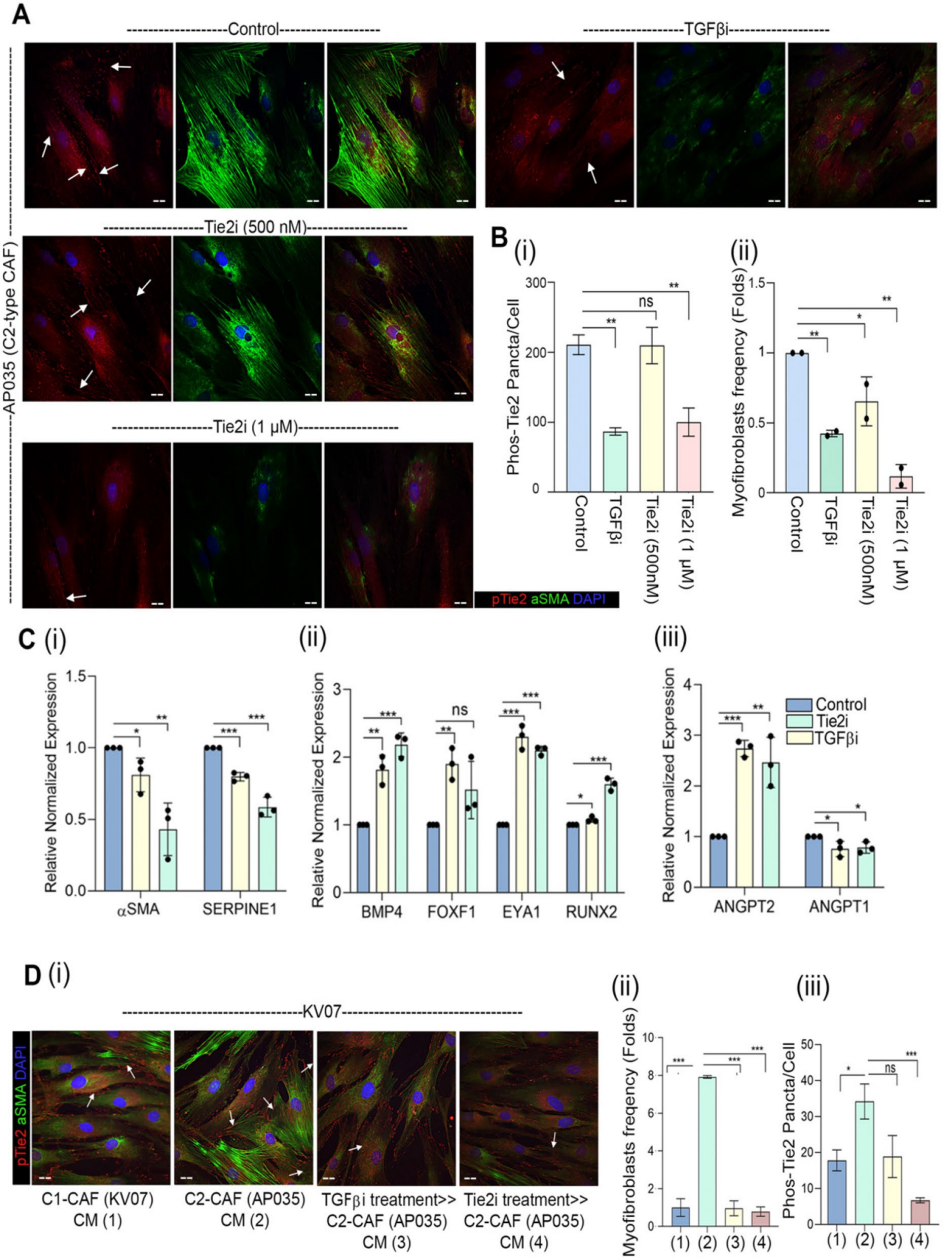
Tie2-activity is regulated in an autocrine manner.(**A**) Representative images of constitutively active C2-CAF (AP035) detected for αSMA and pTie2 (Y992) protein. Increasing doses of Tie2 inhibitor and TGFβ inhibitor (Galunisertib; 1µM) were used to block respective receptor activity. Cells were quantified using ImageJ software. (**B**) (i) quantification of pTie2 (Y992) puncta and (ii) myofibroblasts frequency under these conditions. (**C**) qPCR analysis of (i) C2-CAF related genes (*SERPINE1*, α*SMA*), (ii) C1-CAF related genes (*BMP4*, *EYA1*, *RUNX2*, *FOXF1*), and (iii) ligand of Tie2 receptor (*ANGPT1*, *ANGPT2*) following Tie2 inhibitor and TGFβ inhibitor treatment in constitutively activated C2-CAF. Unstimulated CAF in same media was used as control. (**D**) (i) Representative images of C1-CAF (KV07) exposed to conditioned media from C1-CAF (KV07), C2-CAF (AP035), TGFβ inhibited C2-CAF (TGFβi > C2 CAF), Tie2 inhibited C2-CAF (Tie2i > C2 CAF), for 48 h, detected for αSMA and pTie2 (Y992) (ii) myofibroblasts frequency and (iii) pTie2 (Y992) puncta was quantified using ImageJ. Arrowhead indicates pTie2 (Y992) puncta. Scale bar = 20 µm *P < 0.05, **P < 0.01, ***P < 0.001

To strengthen the link between TGFβ and Tie2 in CAF, we next used pharmacological inhibitor of these regulators on a patient-derived C2-CAF (AP035), having constitutive-myofibroblastic phenotype (Fig. 3A). As anticipated, Galunisertib (TGFβi) or Tie2i independently led to reduction in frequency of constitutive phospho-Tie2 (Y992) positive puncta as well as myofibroblast frequency, as compared to control (Fig. 3B, i,ii); suggesting cell autonomous TGFβ receptor activation as cause for constitutive activation of Tie2 in C2-CAF. More interestingly, both Tie2- and TGFβ-inhibited C2-CAF showed significant downregulation of genes associated with C2-CAF (*αSMA* and *SERPINE1*) with concomitant upregulation of genes associated with C1-CAF (*BMP4* and *ANGPT2)* (Fig. 3 C, i-iii); indicating a transition of C2-CAF, back to C1-CAF.

Since both TGFβ and Tie2 signaling are activated through receptor-ligand interactions, we explored if secreted factors from C2-CAF may act as drivers for acquiring and maintaining C2-CAF-like state. Conditioned media of KV07 (C1-CAF) and AP035 (C2-CAF) were collected and put over KV07 (C1-CAF) (Fig. 3D-i). Interestingly, conditioned media of C2-CAF was sufficient to increase both, myofibroblasts frequency (Fig. 3Di) and number of Phospho-Tie2 (Y992)-positive puncta in C1-CAF (Fig. 3Dii). This was significantly reduced when C1-CAF were exposed to conditioned media collected after TGFβ- or Tie2-inhibition in C2-CAF (Fig. 3Di, iii), suggesting the maintenance of Tie2 activity in C2-CAF through autocrine TGFβ signaling.

### TGFβ-induced histone deacetylation drives transcriptional state change associated with C1- to C2-CAF transition

**Fig. 4:**
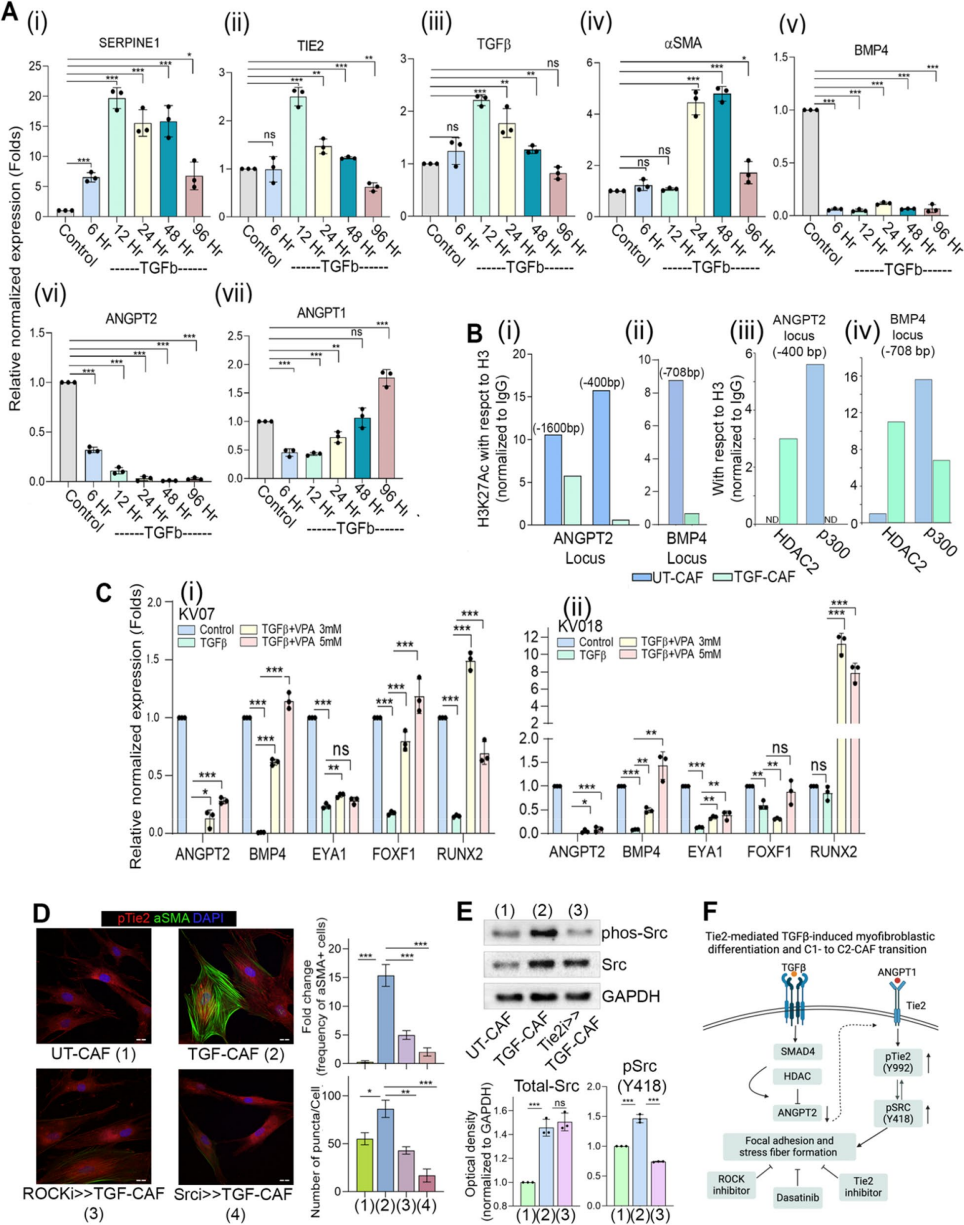
TGFβ-induced histone deacetylation drives transcriptional state changes associated with transition of C1- to C2-CAF.(**A**) (i-vii) qPCR analysis of *SERPINE1,* α*SMA, TGFꞵ, Tie2, BMP4, ANGPT2* and *ANGPT1* in C1-CAF following 10 ng/ml TGFβ stimulation in time dependent manner as indicated. Relative abundance of mRNA was normalized with unstimulated CAF (Control) of respective time points. (**B**) Chromatin immunoprecipitation analysis of H3 K27-acetylation status on (i) ANGPT2 (TATA binding site −1600 bp; initiator site −400 bp) and (ii) BMP4 promoter (−708 bp) in C1-CAF; as well as (iii) HDAC2 and p300 on ANGPT2 initiated (−400 bp) and (iv) BMP4 promoter (−708 bp) locus with 10 ng/ml TGFβ (TGF-CAF). Unstimulated CAF (UT-CAF) were used as control. Data is representative on number of copies detected by ddPCR relative to ChIP DNA for Histone H3. (**C**) qPCR analysis showing expression of C1-CAF related genes, *BMP4*, *EYA1*, *RUNX2*, *FOXF1* and *ANGPT2* with or without valproic acid (3 mM, 5 mM) in presence of 10 ng/ml TGFβ. Unstimulated CAF were used as control. (**D**) Representative images of αSMA and pTie2 (Y992) in UT-CAF and TGF-CAF. ROCK and SRC inhibition was done 1 h before (ROCKi > > TGFCAF or Srci > > TGFCAF) TGFβ-induction. Bar graph showing quantification of myofibroblasts frequency and pTie2 (Y992) puncta, calculated using ImageJ software. (**E**) Western blot analysis of the expression of pSRC and SRC in UT-CAF, TGF-CAF and Tie2i > > TGF-CAF. (**F**) Schematic model of HDAC-mediated suppression of C1-CAF related genes. Scale bar = 20 µm. *P < 0.05, **P < 0.01, ***P < 0.001

To delve into the possible mechanisms, we performed TGFβ-induced gene expression analysis in a timeseries manner (Fig. 4A). Activation of TGFβ-signal increased expression of *SERPINE1* at as early as 6 h which was maximum at 12 h. Expression of endogenous *TGFβ* and *Tie2* genes also showed its peak levels by 12 h of TGFβ-induction. Interestingly, *αSMA* gene showed upregulation only after 48 h; suggesting that Tie2 upregulation preceded *αSMA*-upregulation during myofibroblastic differentiation by *TGFβ*. While genes associated with C2-CAF showed upregulation; we observed very sharp and sustained downregulation of C1-CAF specific genes *BMP4* and *ANGPT2,* at as early as 6 h. Antagonist *ANGPT2* was very significantly suppressed for entire test-period (96 h) of TGFβ induction, agonist *ANGPT1* was significantly upregulated at later time points. Overall, these results indicated the presence of TGFβ-induced feed-forward loop of Tie2-activation by rapid suppression of ANGPT2 followed by upregulation of endogenous *TGFβ*, *Tie2* and *ANGPT1*. Since, our results clearly showed that ANGPT2 was sufficient to block TGFβ-induced myofibroblastic differentiation (Fig. 2C); thus, rapid suppression of ANGPT2 may be one of the most crucial events in TGFβ-induced transition of C1-CAF into C2-CAF. Thus, we next performed chromatin immunoprecipitation to evaluate activation-marks using H3 K27-acetylation for ANGPT2 and BMP4 locus. Interestingly we observed reduced H3 K27-acetylation on TATA binding site (−1600 bp) and initiator site (−400 bp) of ANGPT2 promoter and the tested locus of *BMP4* promoter (−708 bp) in TGF-CAF, compared to UT-CAF (Fig. 4B, i, ii); with concomitant increased binding of histone deacetylase 2 (HDAC2) and absence of acetyl transferase (p300) on the ANGPT2 and BMP4 initiator/promoter locus upon TGFβ-induction (Fig. 4B, iii, iv). Next, using three different C1-CAF, we tested the effect of TGFβ-induction in presence of potent histone deacetylase (HDAC) inhibitor, Valproic acid (VPA). Suppressive effect of TGFβ on all tested C1-CAF associated genes, *BMP4, EYA1, FOXF1, RUNX2* and *ANGPT2* were significantly much lower, in presence of VPA (Fig. 4C).

In order to explore the possible mechanism of Tie2-signalling in mediating the TGFβ-induced myofibroblastic differentiation, we explored the known players in the process. As anticipated, inhibition of Src kinase activity by a potent inhibitor Dasatinib [43], or the ROCK activity by Y27632; both resulted in significant loss of stress fiber formation as well as Tie2-phophorylation, clearly demonstrating their regulatory role in TGFβ-induced Tie2-activity (Fig. 4D). Interestingly, Tie2-inhibition significantly reduced the phosphorylation of Src (Y418) (Fig. 4E), indicating the crucial role for Tie2 in TGFβ-induced Src-activation. Therefore, Tie2 may reciprocally activate Src, and may serve as novel mediator of TGFβ-induced stress fiber formation in myofibroblasts. Thus, overall as one of the possible mechanisms, the TGFβ-induced deacetylation of open chromatin on C1-CAF associated genes including *ANGPT2*, led to the activation of Tie2-Src-ROCK circuit during myofibroblastic differentiation and transition into C2-CAF (Fig. 4F).

### Endogenous TGFβ is necessary and sufficient for driving Tie2-ANGPT signalling

**Fig. 5:**
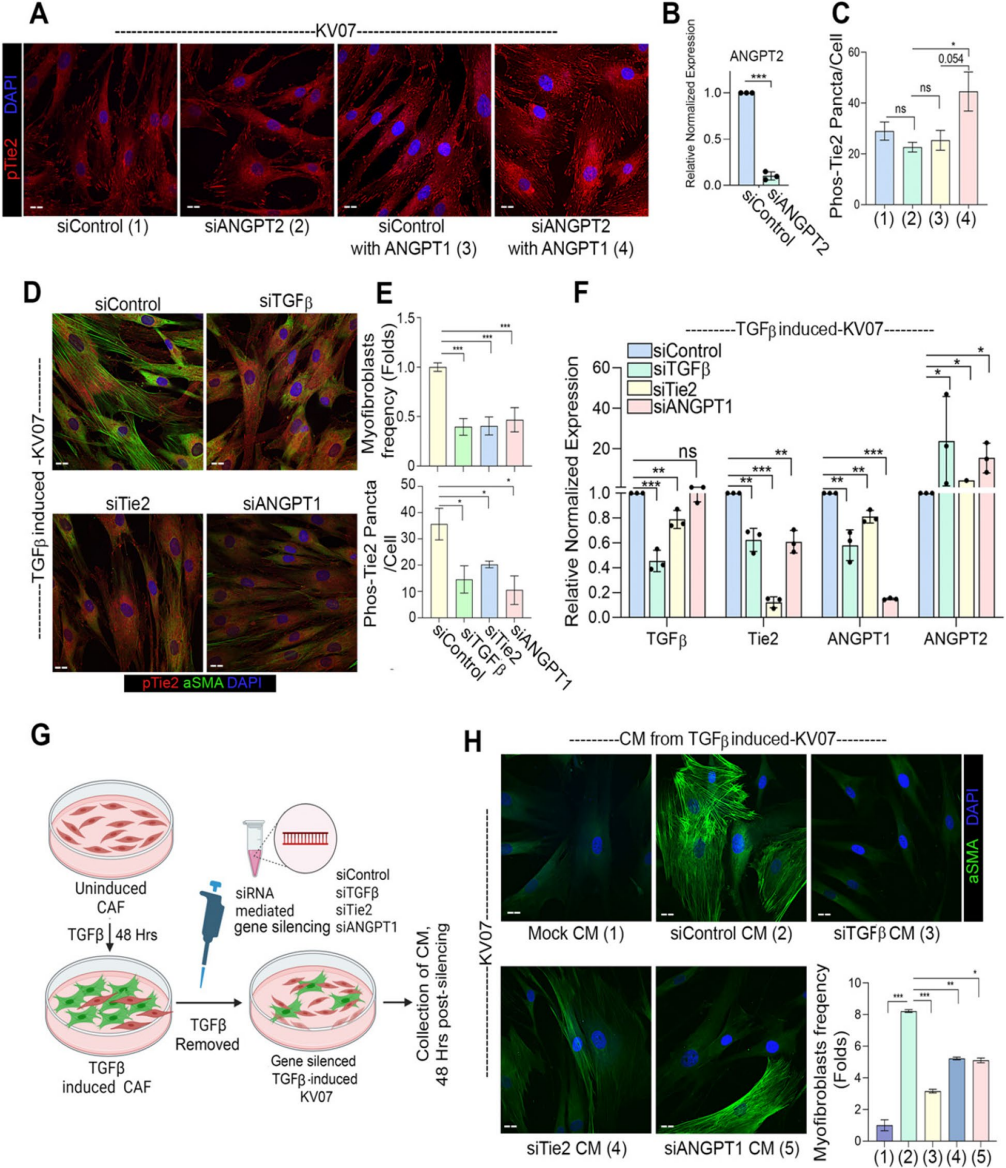
Endogenous-TGFβ is necessary and sufficient in driving Tie2-ANGPT signaling.(**A**) Representative images of ANGPT2 silenced C1-CAF with or without ANGPT1 stimulation (400 ng/ml) for 6 h, detected for pTie2 (Y992) protein. Scrambled siRNA was used as control. (**B**) qPCR analysis of *ANGPT2* following *ANGPT2* knockdown in C1 CAF. (**C**) Quantification of pTie2 (Y992) puncta using ImageJ. (**D**) Representative images of αSMA and pTie2 (Y992) protein detected by immunofluorescence staining, upon gene silencing of TGFβ, Tie2 and ANGPT1 in TGF-CAF. Scrambled siRNA was used as a control. (**E**) Myofibroblasts frequency and pTie2 (Y992) puncta was quantified using ImageJ. (**F**) qPCR analysis of *TGF*β*, Tie2, ANGPT1* and *ANGPT2* followed by knockdown of *TGF*β*, Tie2* and *ANGPT1* in TGF-CAF. (**G**) Schematic model suggesting experimental design of conditioned media (CM) collection from TGF-CAF following *TGF*β, *Tie2* and *ANGPT1* gene knockdown. (**H**) Representative images showing myofibroblasts frequency in uninduced C1-CAF exposed to the CM collected from TGF-CAF after TGFβ, Tie2 or ANGPT1 gene-silencing. C1-CAF exposed to C1-CAF CM was used as control. Myofibroblasts frequency was quantified using ImageJ. Scale bar = 20 μm. *P < 0.05, **P < 0.01, ***P < 0.001

Since TGFβ-induced suppression of *ANGPT2* was found important for Tie2-phosphorylation and myofibroblastic differentiation, we next explored if downregulating endogenous ANGPT2 may be sufficient for Tie2-phosphorylation in oral CAF. As such, ANGPT2 silencing in C1-CAF did not result in any significant change in pTie2 (Y992)-positive puncta (Fig. 5A); however, addition of ANGPT1 increased the number of phosphorylated-Tie2 puncta in ANGPT2-silenced C1-CAF (Fig. 5B-C). Thus, downregulation of ANGPT2 was necessary for ANGPT1 induced Tie2-phosphorylation in oral CAF. Since, TGFβ-induced CAF showed upregulation of endogenous-TGFβ, Tie2 and ANGPT1 along with suppression of ANGPT2, we next silenced increased levels of TGFβ, Tie2 or ANGPT1 in TGF-CAF. As anticipated, Tie2 and ANGPT1 silencing resulted in decreased pTie2 (Y992) levels and reduced frequency of myofibroblasts in TGF-CAF (Fig. 5D, 5E). Interestingly, even silencing of upregulated endogenous-TGFβ also suppressed Tie2-phosphotylation; supporting the role of endogenous-TGFβ in maintaining myofibroblast phenotype, as observed with the constitutive C2-CAF (Fig. 3). Silencing of respective genes was confirmed by qPCR (Fig. 5F). Importantly, reducing the levels of all three genes showed increased expression of ANGPT2 in TGF-CAF, suggesting it to be in an interconnected regulatory signaling loop (Fig. 5F).

Since, there was an increase in ANGPT2 expression after silencing of induced levels of *TGFβ, Tie2* or *ANGPT1* in TGF-CAF, this prompted us to test if conditioned media (CM) from these experiments may show reduced myofibroblastic differentiation potency (Fig. 5G). As anticipated, conditioned media from control-siRNA transfected TGF-CAF was sufficient to significantly increase the myofibroblasts frequency in UT-CAF. However, UT-CAF, when exposed to CM collected from *TGFβ, Tie2* or *ANGPT1* siRNA transfected TGF-CAF showed significantly lower frequency of myofibroblasts (Fig. 5H). Collectively, these results led us to conclude that either extrinsic or endogenous-TGFβ in oral CAF led to the activation of Tie2-ANGPT signal; possibly as one of the most responsible factors for transitioning of C1-CAF to C2-CAF and acquiring myofibroblast phenotype.

### C1-CAF or C2-CAF derived gene expression signatures showed concordance respectively with BMP4-High and ITGA3-High CAF, in situ

**Fig. 6:**
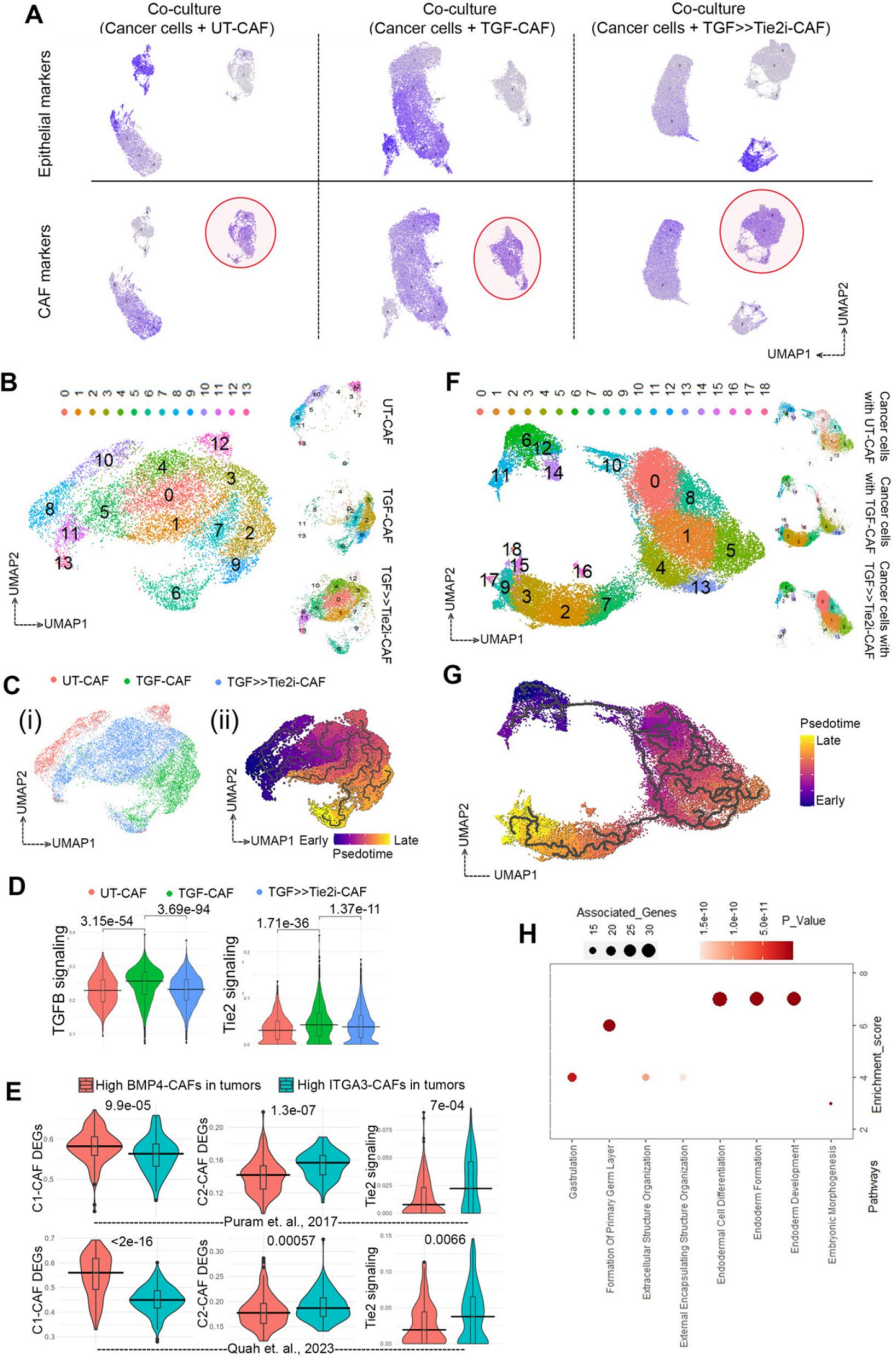
TGFβ-induced myofibroblastic C2-CAF reprograms oral-cancer cells to acquire embryonic-like transcriptome state.(**A**) Feature plot showing expressions of epithelial and CAF marker modules on UMAP projection from three conditions of co-cultures as indicated. Circled clusters are annotated as CAF clusters with high CAF marker module scores and negativity for epithelial markers module scores. (**B**) UMAP plot shows re-clustering of CAF clusters from all the three conditions merged, revealing 13 clusters with a total of 11,391 cells. A split view of major clusters in a sample specific manner is provided on side panel. (**C**) (i) An UMAP plot visualizing sample wise grouped CAF clusters. (ii) Monocle3 pseudotime -time analysis showing CAF dynamic transition along the trajectory. (**D**) Violin plot showing enrichment of TGFβ and Tie2 signaling AUC scores generated by R tool ‘AUCell’, upon TGFβ treatment of CAF, which was significantly decreased followed by Tie2-inhibition. (**E**) AUC scoring of CAF from classified patient groups (High BMP4 (C1-like)/High ITGA3 (C2-like)) from Puram et al. and Quah et al. HNSCC datasets shows significant enrichment of C1-CAF DEGs in High BMP4 group, and C2-CAF DEGs and Tie2 signaling in High ITGA3 group. (**F**) Subset of 32,354 epithelial cells from all three conditions were merged and re-clustered, identified 16 clusters, projected on UMAP plot. (**G**) Pseudotime analysis exploring transition trajectory of cancer cells. (**H**) Bubble plot showing GO biological process analysis of gene-set among single cell and bulk RNA sequencing of cancer cells co cultured with TGF-CAF. Size of bubble represents numbers of associated genes and colour corresponds to given *p* value

To get deeper understanding about the intricate interplay between different CAF-phenotypes and their influence on oral cancer cells, we performed single cell RNA sequencing (scRNAseq) for co-cultures of oral cancer cells with C1-CAF (UT-CAF) or TGFβ-induced CAF (TGF-CAF) or TGFβ-induced-Tie2-inhibited (TGF > > Tie2i-CAF), separately (Figure S4 A, B (i,ii,iii)). Based on module scores of canonical markers of epithelial (*KRT5, KRT14, KRT17*) and CAF (*FAP, THY1, PDGFRA, PDPN*) related genes from gene-set [44], we identified CAF clusters from each of the co-culture conditions, having high-scores for CAF gene-set and low score for cytokeratin enriched epithelial gene set (Figure S5 A, Fig. 6 A). Unsupervised re-clustering of segregated 11,391 CAF from 3 different conditions (Fig. 6B) showed transcriptional divergence on UMAP projection acquiring three distinct transcriptional states. ‘Pseudotime analysis’ performed using ‘Monocle3’, demonstrated the origin of TGF-CAF from UT-CAF at significant scale and depth; whereas, TGF > > Tie2i-CAF displayed a retrogressive transcriptional behaviour to remain in middlemost part of trajectory indicating a reversal of TGF-CAF towards UT-CAF upon Tie2-inhibition (Fig. 6C (i, ii), S5B). Clusters belonging to UT-CAF such as 10,8,11,13 had a lower pseudotime value than that of clusters comprises of TGF-CAF and TGF > > Tie2i-CAF, depicting a continuous evolution of CAF phenotypes from UT CAF to TGF-CAF through TGF > > Tie2i-CAF (Figure S5B). Next, we performed pseudo-bulk analysis of scRNAseq data to evaluate the cell-state specific combined features; where individual UT-CAF (Red), TGF-CAF (Green) and TGF > > Tie2i-CAF (Blue) were computed for ‘AUCell scores’ for TGFβ- or Tie2-signaling associated gene-sets as signatures. Interestingly, it showed significantly higher score for both signature in TGF-CAF with significant downregulation in TGF > > Tie2i-CAF (Fig. 6D, S5 C-i,ii). This clearly supported the reversal of C2-CAF towards transcriptional state of C1-CAF, upon Tie2-inhibition, as observed from pseudotime analysis.

To evaluate the presence of C1-type and C2-type CAF in patient derived samples, we investigated the single cell transcriptome datasets of treatment-naive HNSCC tumors from two independent earlier studies by Puram et. al. and Quah et. al. [44, 45]. Based on the consistent differential expression of BMP4 and ITGA3 in our current datasets and previously reported study from our group [15], we considered BMP4 and ITGA3 as markers for C1-type and C2-type CAF, respectively. Patients with higher than median expression score of BMP4 and concomitant lower than the median expression score for ITGA3 were classified as High-BMP4-CAF patients. Vice versa, individual patients with higher than the median expression score of ITGA3 and lower than the median expression score for BMP4 were classified as High-ITGA3-CAF patients (Supplementary Figure S6 A, B, C). Crucially, the AUCell scoring was performed for the classified patient groups using scRNAseq-DEGs between our UT-CAF and TGF-CAF (Adj. *P* value < 0.05) (Table S3). Validating the classification, CAF in high-BMP4-CAF patient group showed significantly higher score for upregulated UT-CAF-DEGs, whereas; CAF in high ITGA3-CAF patient group showed significantly higher score for upregulated TGF-CAF-DEGs (Fig. 6E), for both Puram et. al. and Quah et. al. studies. Furthermore, in both these studies, CAF in high-ITGA3 group patients showed significantly higher score for Tie2 signaling, aligning with our observation of Tie2 pathway enrichment in TGF-CAF or C2-CAF derived from patients (Fig. 6E).

### CAF-specific Tie2 activity regulates cancer cell plasticity and stemness in oral cancer cells

Previously we have reported that myofibroblastic C2-CAF drives stemness in oral cancer cells [15]. Therefore, TGFβ induced Tie2-signal in CAF might act as a potential target against C2-CAF driven cancer cell reprogramming. Thus, we next performed deeper investigation on the cancer cell reprogramming ability of Tie2-activity in C2-CAF by evaluating the transcriptome state of cancer cells using our co-culture derived scRNAseq data. A total of 32,354 epithelial cells were clustered together from all the conditions to broaden our knowledge on how different subtypes of CAF modulate cancer cell transcriptome (Fig. 6F). Re-clustering patterns of cancer cells revealed 3 major clusters with a total of 18 sub-clusters encompassing different transcriptional states (Figure S7 A-i,ii,iii) 6 F). While the one major subset of clusters (clusters 6,10–12,14) was common in all three conditions; surprisingly we observed other sets of cancer cells (clusters 0,1,4,5,8,13) shared majorly common clustering neighbourhood when co-cultured with UT-CAF or with TGF > > Tie2i-CAF, suggesting close similarity in their gene expression patterns. Interestingly, a very distinct subset of clusters (clusters 2,3,7,9,15–18) was comprised of cancer cells from TGF-CAF coculture (Table S4), depicting TGF-CAF induced transcriptional reprogramming of cancer cells, which was apparently absent when cancer cells were co-cultured with Tie2-inhibited TGF-CAF (Fig. 6F). Pseudotime analysis suggested a dynamics of cancer cell transition trajectory, highlighting that upon co-culture with TGF-CAF this subset of oral cancer cell acquired more evolved state on transition-axis with respect to clusters which were unchanged in any co-culture conditions (Fig. 6G, S7B). Further, relative position of cancer cells in co-culture with UT-CAF and with TGF > > Tie2i-CAF were almost indistinguishable in axis, implying that Tie2-inhibition in C2-CAF suppressed the cancer cells reprogramming ability of C2-CAF.

Emergence of this unique transcriptionally reprogrammed subset of cancer cells upon co-culture with TGF-CAF prompted us to further characterize their molecular nature. We overlapped the differentially upregulated genes in this unique subset of cancer cells (clusters 2,3,7,9,15–18) with genes which were differentially upregulated in cancer cells co-cultured with TGF-CAF in comparison to UT-CAF from our bulk-RNAseq data (Fig. 1). 150 DEGs were identified as common among both lists; majorly harboured biological process of early developmental processes, indicating an embryonic-like reprogramming of cancer cells by TGF-CAF (Fig. 6H, S7 C, Table S5, S6). Taken together, our data clearly suggested that TGFβ induced myofibroblastic C2-CAF, reprograms oral-cancer cells to acquire an undifferentiated phenotype which may have more aggressive functions.

**Fig. 7:**
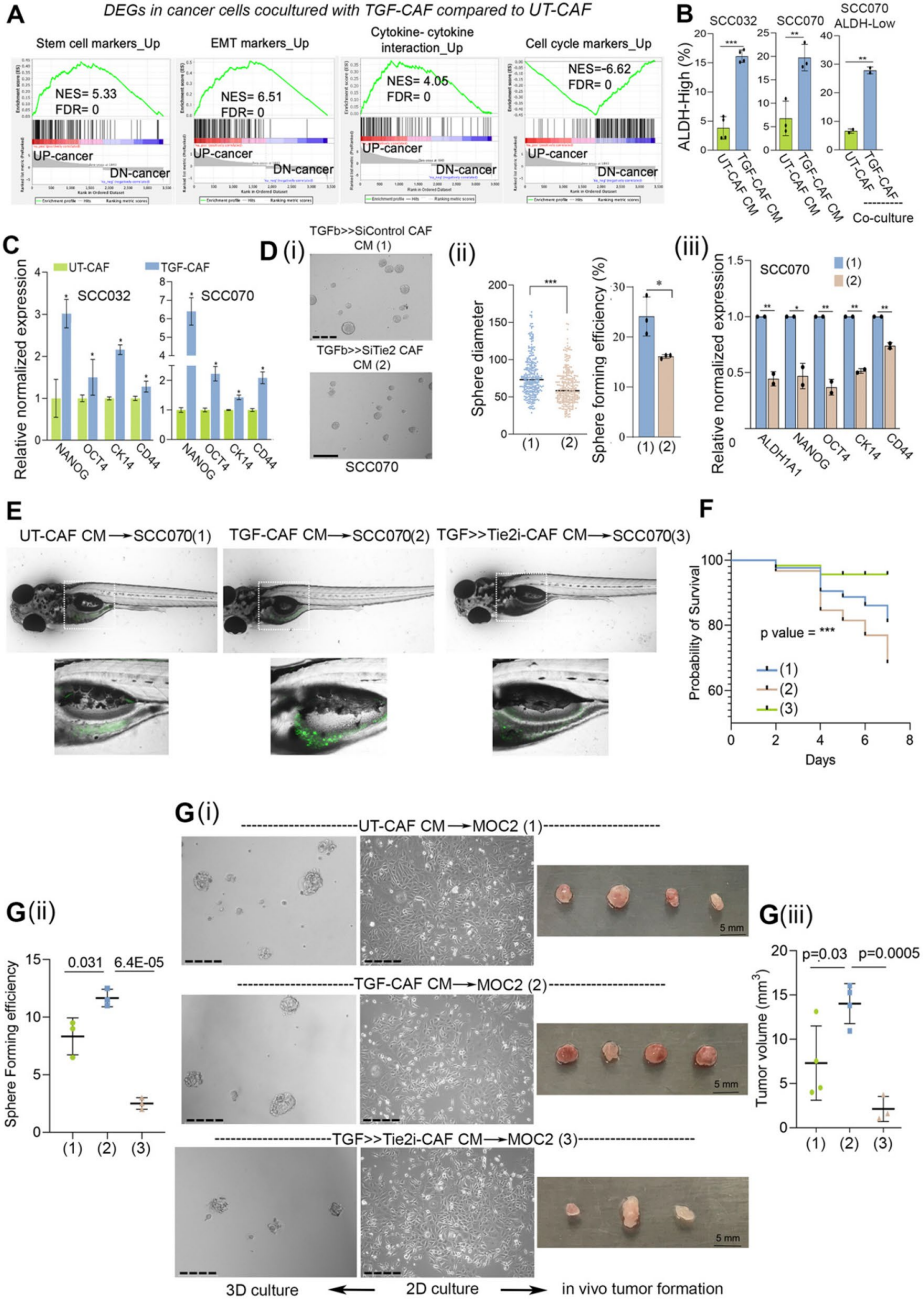
CAF-specific Tie2 regulates cancer cell plasticity and stemness in oral cancer cells.(**A**) Gene set enrichment analysis (GSEA) from transcriptome data of cancer cells, co-cultured with UT-CAF or TGF-CAF for four days. Datasets were obtained from MsigDB database. (**B**) Bar graph showing conversion of ALDH-Low cells into ALDH-High cells upon exposing to conditioned media of UT-CAF or TGF-CAF or upon co-culture as indicated. **(C)** qPCR analysis of stemness associated genes (*OCT4*, *NANOG*, *CD44* and *KRT14* (*CK14*) in two different oral cancer cell lines (SCC070 and SCC032) exposed to CM from KV07 or KV018 CAF, respectively. (**D**) (i) Representative image of 3D-spheroids of SCC070 cell line exposed to CM from TGFβ > > siTie2 or TGFβ > > siControl, followed by testing in spheroid formation assay. (ii) Dot plot showing diameter of formed spheroids of cancer cells from these conditions and bar graph showing sphere forming efficiency of cancer cells exposed to both these conditions. Sphere size was quantified using ImageJ. Spheres of < 60 µm diameter were excluded from study. (iii). qPCR analysis of stemness associated genes (*ALDH1 A1*, *OCT4*, *NANOG*, *CD44* and *KRT14/CK14*) in cancer cells following exposed to CM from TGFβ > > siControl or TGFβ > > siTie2 in monolayer culture for 48 h. (**E**) Representative images of zebrafish xenografts taken using confocal microscope. GFP positive oral cancer cells (SCC070) were exposed to conditioned media of UT-CAF, TGF-CAF or TGF > > Tie2i-CAF for 48 h. Cells were harvested and 100 cells were inoculated into yolk sac of each zebrafish embryo (2-day post fertilization). GFP-positive cell colonies were visible on 4 th day of inoculation. (**F**) Kaplan Meier survival plots showing a probability of deaths in zebrafish embryos due to increased tumor burden. (**G**) (i) Representative phase contrast images of MOC2 cells cultured with conditioned media of UT-CAF, TGF-CAF and TGF > > Tie2i CAF for 48 h in monolayer culture (2D) and representative images of 3D spheroids of MOC2 cells exposed to CAF-CM from all three conditions as mentioned. (ii) Tree plot showing sphere forming efficiency of MOC2 cells exposed to conditioned media of UT-CAF, TGF-CAF and TGF > > Tie2i CAF. Spheres of < 60µ diameter were excluded from study. (iii) MOC2 cells cultured in conditioned media of UT-CAF, TGF-CAF and TGF > > Tie2i CAF for 48 h in monolayer culture. These CM exposed MOC2 cells (3 × 10^5^ cells/mice) were subcutaneously inoculated into syngeneic C57BL/6 mouse models and monitored for 10 days. On day 10 th of transplantation, mice were sacrificed and tumors were harvested. Volume of these tumors were measured using ImageJ and plotted in GraphPad prism. **P < 0.05, **P < 0.01, ***P < 0.001. Scale bars, 275 µm

Our bulk-RNAseq analysis revealed a total of 1843 and 1568 genes upregulated and down-regulated respectively in cancer cells co-cultured with TGF-CAF compared to UT-CAF (Table S7). GSEA analysis identified enrichment of signatures for stem cell, EMT, cytokine-cytokine interaction and downregulation of cell-cycle in cancer cells co-cultured with TGF-CAF group (Fig. 7A). In support of obtained downregulation of cell cycle marker gene-set; frequency of Ki67-positive cells was found to be reduced in cancer cells co-cultured with TGF-CAF, as compared to UT-CAF (S8 A-i, ii). Previous data from our lab has demonstrated an increased frequency of ALDH^High^ stem-like cancer cells (SLCCs) in co-culture with C2-type CAF [15]. Similarly, significantly higher frequency of ALDH^High^ phenotype was observed when oral cancer cells were exposed to TGF-CAF CM (Fig. 7B, S8B). Recently we have revealed plasticity in oral cancer cells having ALDH^High^ and ALDH^Low^ phenotype [46]. This instigated us to sort ALDH^Low^ cells and coculture with UT-CAF and TGF-CAF for four days. Results clearly suggested that TGF-CAF can significantly favour the shift of ALDH^Low^ cells into ALDH^High^ cells (Fig. 7B, S8 C). Further, gene expression of cancer cells showed upregulation of stemness associated genes *NANOG*, *OCT4*, *CK14* and *CD44* in two different cancer cell lines exposed to CM of TGF-CAF compared to that of UT-CAF (Fig. 7C); suggesting the possibility of induction of stemness in cancer cells by TGF-CAF.

So far, transcriptome data suggested an ability of TGF-CAF in educating cancer cells to acquire more aggressive transcription state which was significantly suppressed after inhibition of Tie2 activity in CAF. Therefore, this possibility was next evaluated against stemness in cancer cells by targeting Tie2 expression and activity in TGF-CAF. Oral cancer cell (SCC070) exposed to CM collected from TGF-CAF transfected with Tie2-siRNA (TGF > > siTie2-CAF) showed significant downregulation of tested stemness related genes *NANOG, OCT4, ALDH1 A1, CK14* and *CD44* (Fig. 7D) as well as spheroid forming efficiency, as compared to CM collected from CAF transfected with control-siRNA (TGF > > siControl-CAF) (Fig. 7D, i-ii). Similar results were obtained with sphere forming efficiency of two different oral cancer cell lines. This was significantly increased when exposed to conditioned media of TGF-CAF compared to control (UT-CAF); whereas it was suppressed when exposed to CM from TGF > > Tie2i-CAF in both the tested cell lines (Figure S9 Ai-ii, S9B) without showing any effect of growth properties of cells growing in adherent condition with serum. Suggesting the reduced 3D-spheroids growth to be an indicator of affected stemness in cancer cells (Figure S9 C). Together, our scRNAseq data analysis and cellular functional assays strongly supported the notion that TGF-CAF-expressed Tie2 may play one of the most crucial role in driving cellular plasticity and maintaining higher stemness in oral cancer cells.

We next evaluated the impact of CAF-induced cancer cell reprogramming on tumor forming ability of oral cancer cells. First, GFP expressing SCC070 oral cancer cell line was exposed to CM obtained from UT-CAF, TGF-CAF or TGF > > Tie2i-CAF for 48 h. Cells were harvested and 100 cells were injected into yolk sac of each 2 dpf (two days post fertilization) embryo. Cancer cell foci formation was monitored under fluorescent microscope for up to seven days and mortality was recorded. Confocal images were taken after 4 days post injection of cancer cells. Interestingly, similar to the results obtained with sphere formation; SCC070 cells incubated with CM of TGF-CAF showed maximum tumor foci formation and also highest mortality of embryos (Fig. 7E, F). Interestingly, embryos injected with SCC070 exposed to CM of UT-CAF and TGFβ > > Tie2i-CAF did not show significant cancer cell foci formation within the tested time period (Fig. 7E). Importantly, we observed better survival of embryos injected with cancer cells which were exposed to CM of TGFβ > > Tie2i-CAF (Fig. 7F). Encouraged from these observations, we next aimed to perform tumor formation assay using murine syngeneic mouse model of oral cancer. Towards this, we first tested if human-CAF-derived CM may exert similar effect on C57BL/6 mouse oral cancer derived cell line, MOC2. Very interestingly, similar to human oral cancer cell lines, sphere forming efficiency of MOC2 cells was significantly increased when exposed to conditioned media of TGF-CAF compared to control and suppressed when exposed to CM from TGF > > Tie2i-CAF (Fig. 7G, i-ii); without showing any effect on growth of adherent cell culture with serum (Fig. 7G-i). Next, MOC2 cells (3 × 10^5^ cells/mice) exposed to different CM were allografted subcutaneously into wild-type C57BL/6 mice. Significantly higher tumor volume was observed in conditions where MOC2 cells were exposed to CM of TGF-CAF compared to UT-CAF. In contrast, only 3 out of 4 animals developed tumor and volume of developed tumor was significantly lesser for allografted MOC2 cells exposed to CM of TGFβ > > Tie2i-CAF (Fig. 7G-iii). Overall, data clearly supported the possible impact of Tie2 activity in TGF-CAF, driving cell state transitions of oral cancer cells to acquire stemness.

### Tie2 responsive single cell gene expression data derived modules translate to clinical output of HNSCC patients

**Fig. 8:**
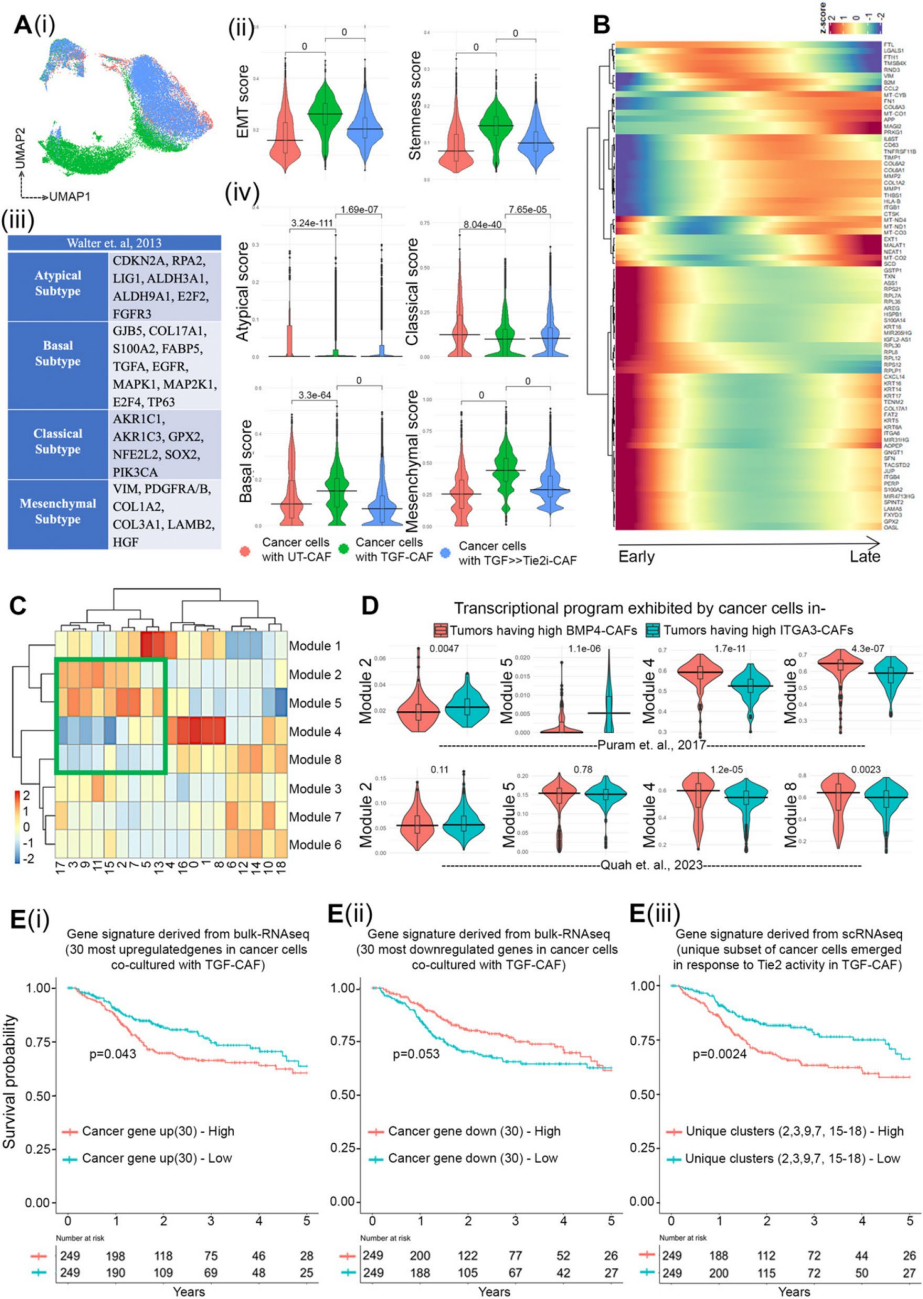
Tie2 responsive single cell gene expression data derived modules translate to clinical output of HNSCC patients.(**A**) (i) UMAP plot showing colour coded clustering of cancer cells co-cultured with UT-CAF, TGF-CAF and TGF > > Tie2i-CAF. (ii) Violin plot showing EMT and stemness AUC score generated by R tool ‘AUCell’. (iii) Table depicting gene expression based classified molecular subtypes of HNSCC signatures (from [47]) (iv) Pseudo-bulk analysis of AUCell scores over cancer cells co cultured with distinct CAF subtypes for the given molecular subtype gene signatures. (**B**) Heatmap showing trajectory variable gene expressions from early to late pseudotime. (**C**) Expression heatmap of co-regulatory gene modules for each cluster of merged cancer cell subset. Marked green box indicates similar expression pattern of module 2, module 5; and module 4, module 8 on exclusive Tie2 responsive cancer cell clusters. (**D**) AUC scoring of cancer cells from the aforementioned patient groups from Puram et al. and Quah et al. HNSCC datasets shows significant enrichment of modules 4 & 8 in High BMP4 group (C1-like CAF high tumors) in both datasets, and modules 2 & 5 in High ITGA3 group (C2-like CAF high tumors) in Puram et al. and Quah et. al. dataset. (**E**) Prediction of survival probability of TCGA HNSCC patients. Kaplan Meier plot showing survival probability of HNSCC patients harbouring gene signatures of (i)Top 30 upregulated or (ii)Top 30 downregulated genes of TGF-CAF cocultured cancer cells form bulk RNAseq data (iii) Survival probability of patients harbouring gene expression signature obtained from scRNAseq analysis of unique subset of cancer cells co-cultured with TGF-CAF, as mentioned

Emergence of more aggressive transcriptome state due to the dynamic influence of interaction between the CAF-subtypes and co-cultured cancer cells, prompted us to evaluate the translatability of our observed in vitro cellular processes for its clinical significance. The deconvoluted scRNAseq data, where individual cancer cells cocultured with UT-CAF (Red), TGF-CAF (Green) and TGF > > Tie2i-CAF (Blue) were first computed for AUCell scores of EMT and stemness signature (Fig. 8A-i, S10 A-i, ii). As anticipated, this pseudo-bulk analysis of data indicated that cancer cells in co-culture with TGF-CAF are enriched with EMT and stemness related genes, which significantly reduced when cancer cells were co-cultured with TGF > > Tie2i-CAF signatures. Thus, we next mapped these cellular states with the expression signatures of four previously reported molecular subtypes of HNSCC, namely atypical, basal, mesenchymal, classical (Fig. 8 A-iii) [47, 48]. While cancer cells in all different conditions showed very low score for atypical subtype signature; cancer cells cocultured with the UT-CAF (Red) showed significant but marginally higher AUCell score for the classical subtype gene signatures. However, TGF-CAF cocultured cancer cells (Green) had highly significant enrichment of cells with expression pattern for the basal and mesenchymal subtype genes; as shown by AUCell scores (Fig. 8 A-iv, S10B). Very interestingly, cocultured cancer cells with TGF > > Tie2i-CAF (Blue), retained lower expression of basal and mesenchymal genes signature. Thus, Tie2 activity in TGF-CAF may drive basal/mesenchymal subtype program in oral cancer cells; however, we will need to perform more experiments to test this hypothesis which is beyond the scope of this manuscript.

Next, we explored the underlying key transcriptional networks, as drivers of TGF-CAF-induced cancer cell reprogramming in response to Tie2 activity. We obtained differentially expressed, trajectory variable genes that changed over the pseudotime shown in Fig. 6G (Fig. 8B). Using this differentially expressed gene set, we constructed coregulatory gene modules of the cancer cells, resulted in eight dynamically regulated gene modules across all single cell clusters of cancer cells. Very interestingly, modules 2 and 5 were collectively upregulated and modules 4 and 8 were downregulated in cluster 2,3,5,7,9,11,13,15, and 17 (Fig. 8C). Interestingly, except cluster 5 and 11 all these clusters were mainly contributed by subsets of oral cancer cells in response to Tie2 activity in TGF-CAF. The downregulated modules (4 and 8) showed regulation of translational process and upregulated module (2 and 5) showed the process of cell junction organization and cell migration (Figure S10 C).

To explore if these modules are operated in cancer cells in situ in presence of C1-type and C2-type CAF within the oral tumors; we utilized our previously classified patient groups (Figure S6 A, B,C) and evaluated the single cell gene expression pattern of malignant cell population in primary tumor from two independent studies done by Puram et. al. and Quah et. al. [38, 39] (supplementary Figure S10 D,E). Dimensional reduction of malignant cells subsets from the individual patients in High-BMP-CAF or High-ITGA3-CAF group showed marked difference in the gene expression patterns among these patient groups in UMAP projections (Supplementary Figure S10E). AUCell scoring was performed for this classified patients groups using uniquely expressed genes in modules 2, 5, 4 and 8 (Suppl. Table S8). To our excitement, we observed that the malignant cells from high-ITGA3-CAF patient group showed significantly lower score for modules 4 and 8 for both Puram et. al. and Quah et. al. studies and higher score for modules 2 and 5 in Puram et. al. study (Fig. 8D). Thus, this analysis has provided concordance to our in vitro observation of reprogramming of cancer cell by CAF-specific Tie2-activity, under in situ condition in HNSCC and therefore may have its clinical translatability.

To make clinical interpretation of our observed biology, prognostic significance of the data was next evaluated. We first used DEGs between cancer cells co-cultured with TGF-CAF in comparison to UT-CAF from our bulk-RNAseq data and correlated with expression data of HNSCC patient cohort in TCGA study. Survival analysis was performed using gene-set specific ssGSEA score. Patients with their individual ssGSEA scores, more than mean were classified as'high', and others as'low'. Survival of these groups was estimated using Kaplan–Meier (KM) curves and Cox-regression analyses. Interestingly, among all comparisons (Figure S11 A, B); patients with higher ssGSEA-score for top 30 upregulated genes showed poorer 5-year disease specific survival (Fig. 8E-i); whereas top 30 downregulated genes showed better survival (Fig. 8Ei). Our scRNAseq data has discovered emergence of specific subsets of oral cancer cells with more evolved transcriptome state in response to Tie2 activity in TGF-CAF (Fig. 6G). Therefore, we next tested the gene-set drive from this unique subset of cancer cells, as an alternate signature. Patients with higher ssGSEA-score for this signature also showed significantly poorer 5-year disease specific survival (Fig. 8Eii, S11 C); highlighting the possibility of exhibiting clinical relevance of our observed CAF-specific-TGFβ-ANGPT-Tie2-Src signaling axis-driven reprogramming of oral cancer cells.

## Discussion

Studies on Angiopoietin/Tie2 pathway have been majorly focused on endothelial cell functions, related to angiogenesis and vessel maturation [28, 49]. Here, our work has identified the role of TGFβ-signaling in epigenetic downregulation of *ANGPT2*, leading to Tie2-activation in oral-CAF, with TGFβ-ANGPT-Tie2-Src to be regulating each other in a closed loop. Since, depletion of endogenous TGFβ or Tie2 in primary C2-CAF or TGF-CAF significantly upregulated the levels of *ANGPT2* expression with concomitant decrease in Tie2-phosphorylation and myofibroblast phenotype of CAF, we suggest that TGFβ-induced *ANGPT2* downregulation may be one of the key events in induction of Tie2 signaling and maintenance of C2-CAF state. As one of the possible mechanisms of *ANGPT2* downregulation, we identified the possible role of histone deacetylases 2 (HDAC2) in TGFβ-induced H3k27-deacetylation of the *ANGPT2* and BMP4 promoter. Further, all tested C1-CAF associated genes including *ANGPT2*, showed significantly reduced suppression in presence of inhibitor of class-I HDACs, valproic acid. Supporting our observation, a very recent study has established that the TGFβ/ALK5 driven SMAD (suppressor of mothers against decapentaplegic) 3/4 robustly represses *ANGPT2* by forming a corepressor complex with HDAC5 at the *ANGPT2* promoter in pericytes [50]. Also, TGFβ-induced HDAC7 mediated repression of *PPARGC1 A* gene was found crucial for fibroblasts activation in fibrotic lung tissue [51]. Thus, our data supported by these reports clearly suggest that TGFβ-induced HDAC-activity may play crucial role in Tie2-activation as one of the responsible mediators leading to transition of C1-CAF to C2-CAF and myofibroblastic differentiation.

In endothelial cells, Tie2 signaling activates small GTPase Rac1 through PI3 K and Akt, leading to its localization on adherence junction [52]. However, the mechanisms by which ANGPT-Tie2 signal impacts the formation of focal adhesions, cytoskeleton remodelling and stress fiber arrangement is still under exploration in endothelial cells [53]. It has been known that the non-receptor tyrosine kinase Src activates the Rho-ROCK-FAK circuit during TGF-β-induced maturation of focal adhesion, expression of αSMA and stress fiber formation in myofibroblasts [54–57]. As one of the possible mechanisms, our study is suggesting that Tie2 serves as crucial player in TGFβ-induced Src-phosphorylation (Y418) which may also reciprocally activate Tie2 during stress fiber formation and myofibroblastic differentiation.

CAF as major co-existing component of complex tumor ecosystem, exhibit dynamic molecular interactions to cooperate and co-evolve in tumor microenvironment [58, 59]. Several studies have correlated high abundance of stromal myofibroblastic, αSMA-positive CAF with poor prognosis of oral cancer patients [12, 60–62]; however, studies exploring CAF-driven mechanisms have been limiting in oral cancer. Our previous report had demonstrated the role of myofibroblastic C2-CAF in providing more conducive microenvironment for enhanced stemness [15]. Advancing our understanding; the current study identified CAF-specific Tie2-signaling in reprogramming malignant cells to embryonic cell-like state; suggesting as one of the mechanisms generating stemness-supporting niche in oral tumor microenvironment. Since, CM was sufficient in educating cancer cells and cytokine-cytokine receptor interaction was one of the most significant gene-sets enriched in co-cultured cancer cells; we suggest that secretory factors from Tie2-activated CAF may drive cancer cell reprogramming to acquire stemness in oral tumor. Although, studies have suggested the role of TGFβ-induced CAF in supporting tumorigenic ability of cancer cells [63, 64]; however, further work will be required to identify the specific Tie2-mediated factors secreted from TGF-CAF in driving oral cancer progression.

TGF-CAF showed myofibroblastic phenotype with certain overlapping similarities with CAF-types reported earlier in OSCC tumors. Activation of CXCL9/10/11-CXCR3 axis is shown recently in TDO2^+ve^ myofibroblasts present in OSCCs [65]. Similarly, a recent study performed with T1-stage OSCC tissue, with matched dysplasia and adjacent normal tissue reported a subcluster of CAF as mesen_CAF showing certain resemblance with TGF-CAF; e.g., enrichment of TGFβ, EMT, angiogenesis, and PI3 K-AKT-mTOR pathways [66] or defined classical myofibroblast marker αSMA [44]. Significantly, we have provided evidence of defined CAF-subtypes specific gene signatures as well as Tie2-pathway signature in fibroblast clusters in two independent single cell studies of HNSCC tumors [44, 45]. Thus, the Tie2-induced cellular processes exhibited by TGF-CAF; highlighting the possibility of CAF to undertake endothelial-like transition. Our model system may be appropriate for studying the biology of such transitions in future.

Few clinical trials are being attempted to directly target stromal CAF in solid tumors. Although targeting TGFβ is successful in pre-clinical models, it faced major problems when tested under clinal trials, owing to its dual role [67–69]. Reversal of pro-tumorigenic state by reprogramming CAF using vitamin A and D has been demonstrated [70]. Since, Tie2-active CAF reprogram oral cancer cells to acquire aggressive phenotype; CAF-specific function of Tie2 may provide therapeutic benefit. Supporting this possibility, ‘Rebastinib’; as one of the potent inhibitors of Tie2 is currently under clinical trials against leukaemia and locally advanced and metastatic solid tumors in combination with chemotherapy [71, 72].

Our single cell transcriptome data facilitated us in profiling dynamic changes influenced by interactions between the CAF-subtypes and co-cultured cancer cells. Cancer cells showed enrichment of the signature of mesenchymal/basal-subtype of oral cancer after being reprogrammed by TGF-CAF. Similar malignant-basal subtype specific gene expression was previously found to be positively associated with partial-EM phenotype and negatively associated with differentiation state in malignant cells [44]. In connection to this, the Tie-2 activity in TGF-CAF was found to facilitate co-cultured oral cancer cells to acquire embryonic-like cell state with increased stemness and EMT related gene signatures. Similarly, earlier studies with enrichment of embryonic stem cell signature were correlated with aggressive cancer behaviour and poorer prognosis of oral cancer patients [73–75]. Intriguingly, specific genes which were differentially expressed in subset of cancer cells in response to CAF-specific Tie-2 activity suggested a possible prognostic gene signature in HNSCC patient cohort.

## Conclusion

**Fig. 9:**
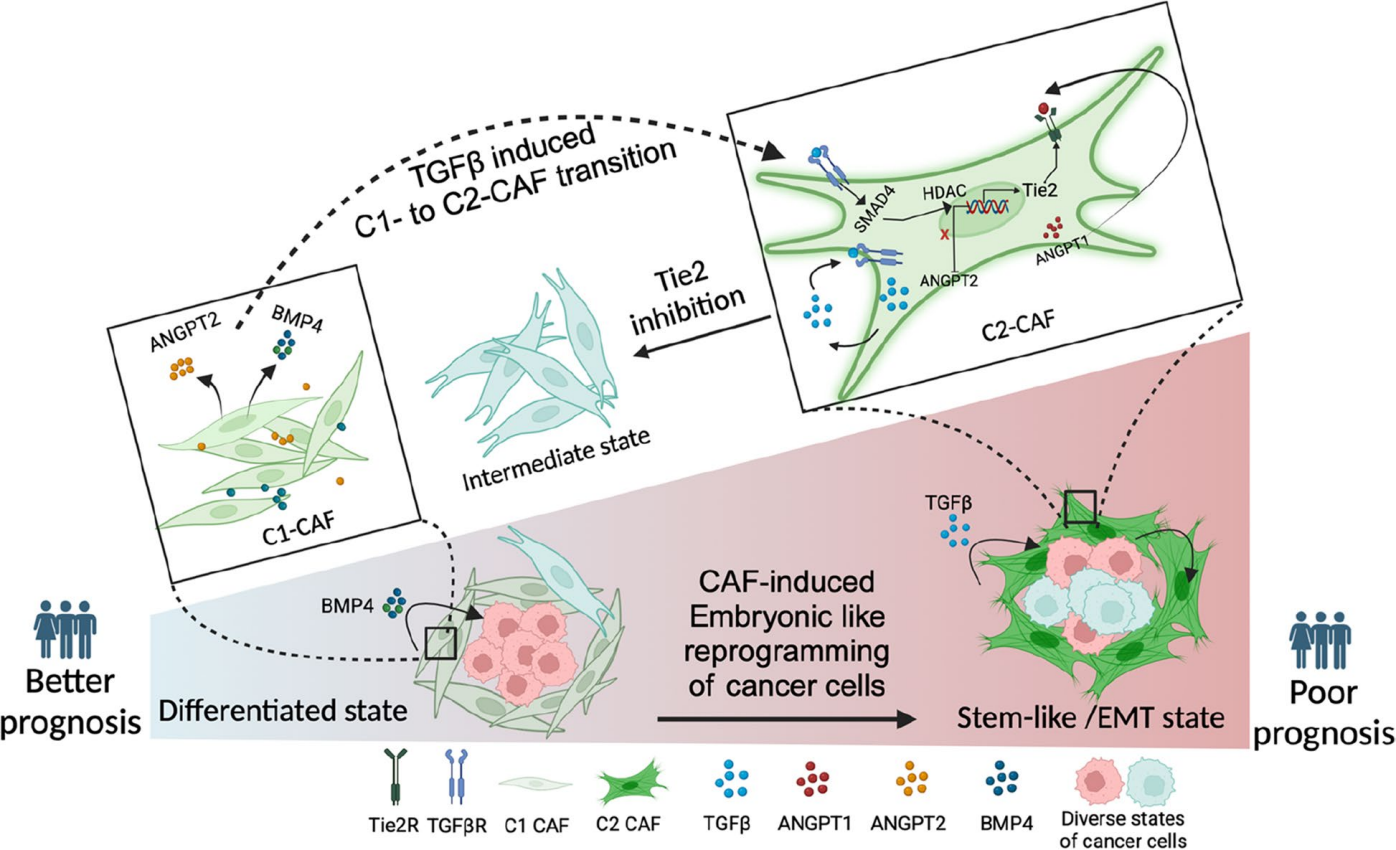
CAF-specific Tie2 activity in reprogramming of oral cancer cells. We have previously identified and characterized C1-CAF and C2-CAF in oral cancer. C1-CAFs exhibit higher-BMP4 expression, whereas C2-CAF exhibit myofibroblastic phenotype with aSMA-positive stress fiber formation. The C2-CAFs supported stem-like properties in cancer cells. Here, we have explored the possible mechanism and demonstrated that the TGFβ-induced myofibroblastic differentiation and conversion of C1-CAF into C2-CAF is mediated through the activation of Tie2-signaling with suppression of its antagonist-ANGPT2 due to HDAC-mediated deacetylation of its promoter. Furthermore, Tie2-inhibition was found to convert TGFβ-induced-CAF towards the transcriptional state of C1-CAF. Functionally, TGFβ-induced CAF reprogramed oral cancer cells into embryonic-like state with enhanced stemness and EMT properties. Emphasizing its clinical translational value, the specific gene-signature derived from the cancer cells, reprogrammed by TGFβ-induced Tie2-activated-CAF, may predict the poor prognosis in head and neck cancer patients

As summarized in Fig. 9, our study has provided the mechanistic evidence of CAF-specific Tie2-signalling as a one of the causal links behind a reported clinical observation where the abundance of myofibroblastic CAF in tumor stroma is associated with poor prognosis in oral cancer patients. Thus, this study is suggesting the possibility of targeting Tie2-signaling as one of the stromal targets in the subset of patients having abundance of myofibroblastic CAF in tumor microenvironment.

### Strength of the study

TGFβ-signaling is successfully targeted in pre-clinical models of cancer but faced many serious problems when tested under clinal trials. Here, our study has provided evidences and arguments for targeting the activated Tie2-sigaling in cancer associated fibroblasts as an alternate approach against the crucial cellular function of TGFβ in oral tumor microenvironment. Moreover, our study has provided validated co-culture models as resource for studying the tumor-stromal interaction in oral cancer progression. Further, the reported gene expression signatures may provide crucial leads for refining molecular subgroups of oral cancer patients for their risk-stratification.

### Limitations of the study

Transplantation of oral cancer cells along with UT-CAF and TGF-CAF requires to be performed in animal models. Possibly, CAF need to be immortalized to survive and genetically modified to maintain its state over several weeks of tumor formation, in vivo. Further, potential effect of Tie2-activated CAF on oral cancer reprogramming directly under in vivo conditions remained to be validated. Other cellular components and extracellular matrix of tumor microenvironment are not explored in the study. Future study should investigate the role of these compositions in cancer cell reprogramming. More experiments to address these limitations will potentially resolve the dynamic process of cell-fate transition leading to aggressive oral cancer behaviour.

## Supplementary Information

Supplementary Material 1.

Supplementary Material 2.

Supplementary Material 3.

Supplementary Material 4.

Supplementary Material 5.

Supplementary Material 6.

Supplementary Material 7.

Supplementary Material 8.

Supplementary Material 9.

## Data Availability

The raw and processed count files for bulk RNA sequencing used in this study are deposited in GEO under accession code GSE294386. 10X scRNAseq raw data and cell ranger output processed files are deposited in GEO and can be accessed from accession code GSE295234. This study didn’t generate unique codes. Codes used to analyze the data are available upon request to the corresponding author. All software and algorithm used in this study are publicly available. Analysed data is provided within the manuscript or supplementary information files.
