# Supplementary material for: Tie2 activity in cancer associated myofibroblasts serves as novel target against reprogramming of cancer cells to embryonic-like cell state and associated poor prognosis in oral carcinoma patients
Source: J Exp Clin Cancer Res. 2025 May 10;44:142. doi: 10.1186/s13046-025-03405-8 (PMC12065280; doi:10.1186/s13046-025-03405-8)
Supplement: Supplementary file 1 — Supplementary Material 1. [file 13046_2025_3405_MOESM1_ESM.pdf]

MATERIALS AND METHODS:

Cell lines and culture conditions:

The human oral cancer cell line SCC070 and SCC032 were obtained from Dr. Susanne M. Gollin, University of Pittsburgh (Pennsylvania). SCC070 cells were maintained in Dulbecco's Modified Eagle Medium (DMEM/F12) medium and SCC032 cells were maintained in Minimal Essential Medium (MEM) and supplemented with NEAA, glutamine and antibiotics and 10% fetal bovine serum. GBC02 and MOC2 were cultured in Epilife and IMDM/F12 media respectively, supplemented with FBS and growth factors. Oral Cancer associated fibroblasts (CAFs) were grown in DMEM/F12 medium supplemented with 10% FBS, 1X ITS and antibiotics. Cells were cultured in an adherent monolayer condition at 37°C under a humidified atmosphere containing 5% CO2. Human subjects were included in this study (EC/GOVT/01/12) after approval obtained from the institutional ethics committee of National Institute of Biomedical Genomics (NIBMG) and the institutional review board of Tata Medical Center (TMC), Kolkata, India.

Establishment of co-culture of CAF and cancer cells:

Cancer associated fibroblasts were co-cultured with oral cancer cells at the ratio of 3:1. At first, CAFs were seeded with 10 ng/ml TGFβ in low serum media (DMEM/F12 supplemented with 1% serum and 0.1X ITS, 1X Antibiotic-antimycotic mix, 37°C, 5% CO_2) for 48 hrs. TGFβ was removed and washed with plain DMEM/F12K media. Cancer cells were added on the top of CAF and co-cultured for 4 days in low serum media. Since, the ratio between CAF and cancer cells is crucial and maintained throughout the study, this co-culture was established fresh for each time of experiment and was not passaged.

Conditioned media generations:

TGFβ induction (10 ng/ml) was done in low serum media (DMEM/F12 supplemented with 1% serum and 0.1X ITS) for 48 hrs. Tie2 inhibitor at given dose was added either before one hour of TGFβ treatment (pre-treatment) or following TGFβ induction (post-treatment) for

indicated time. For all treatment conditions, we seeded approximately 6*10^5 CAF in 100 mm dish and incubated for 48 hrs with respective treatment. Following treatment, cells were washed with plain media and fresh low serum media was added for further 48 hrs. At this point of time CAFs grow to 70-80% confluency. From this culture we collected around 7.5 ml of conditioned media with secretory factors. Collected media was centrifuged at 400g for 5 minutes, supernatant was collected and filtered through 0.22µ filter. In a separate 60 mm dish, 2*10^5 cancer cells were seeded in its respective growth medium and kept for 24 hrs. Subsequently, tumor cells were exposed to 2.5 ml of conditioned media and kept for another 48 hrs for further analysis.

Collagen-I-matrigel contraction assay:

TGFβ induction (10 ng/ml) was done in low serum media (DMEM/F12 supplemented with 1% serum and 0.1X ITS) for 48 hrs. Cells were harvested and mixed with collagen I-matrigel and for another 48 hrs. A matrix of rat tail collagen I (354249, corning) with final concentration of 4.6 mg/ml and matrigel (354234, corning) with final concentration of 2.2 mg/ml was prepared. 1N sterile NAOH (S2770, Sigma) was used to neutralize acetic acid of collagen I-matrigel matrix and diluted in PBS. The gel plug was detached from the sides to allow contraction. Gel plug with no cells was used as control. Matrix contraction was monitored and imaging was done by Chemidoc imaging system (BioRad).

siRNA mediated knockdown:

CAFs were transfected with 50nM of siRNA of Tie2, ACTA2, ANGPT1 and ANGPT2 and scrambled siRNA as a negative control (Eurogentec) for 48 hrs in low serum media using INTERFERin kit (Cat# 409-10; Polypus) following manufacturer's instructions.

Lentivirus transduction:

The PLenti.CMV.GFP.Puro (Addgene#17488) plasmid were transduced into the SCC070 cells. Recombinant Lentivectors were produced by transient transfection of transducing vectors

into HEK293T cells with packaging vectors, a plasmid expressing the HIV-1 gag/pol, tat, and rev genes. Lentiviral vector mediated transduction was validated by GFP reporter expression in fluorescence microscopy and FACS based phenotyping was done to get the pure population of the transduced cells, GFP positive cells were then sorted and expanded.

RNA extraction, cDNA preparation and RT-PCR:

Adherent cells were washed twice with ice-cold PBS and immediately lysed in RLT-plus buffer (Cat. # 74104, Qiagen) containing β-mercaptoethanol. Total RNA was extracted using RNA-Easy plus kit (Cat#74034, Qiagen) as per the manufacturer's instructions. Total RNA was measured in the Nanodrop quantification system (Thermo-Scientific). 500 ng of total RNA was converted to cDNA using iScript advanced cDNA synthesis kit for RT-qPCR (Cat# 1725037, Biorad) according to the instructions of the manufacturer.

The expression level of stemness-related genes was performed in CFX96 real time qPCR machine (Biorad) using iTaq™ Universal SYBR® Green Supermix (Cat# 1725121). The primers were designed using Primer Blast (NCBI). Housekeeping gene GAPDH was used as internal control. The expression levels of mRNAs were normalized to GAPDH (dCT) and the relative normalized expression of test sample was calculated as the fold change relative to the control ($2^{\wedge -}$ddCT).

ALDEFLUOR assay:

The ALDH activity of oral cancer cells co-cultured with CAF was measured using the ALDEFLUOR assay kit (Cat. #01700, StemCell Technology), according to the manufacturer's protocol. DEAB (Diethylaminobenzaldehyde) was used as a negative control for ALDH assay. Anti-human CD90-BV421 antibody (1:50, cat # 562556; BD) was used to stain the CAF and propidium iodide was used to identify non-viable cells. CD90 negative cancer cells were checked for ALDH staining and analysis was done using the FACS-Aria Fusion instrument (Becton Dickinson). Frequency of ALDH high cells were analyzed using FCS express software.

Sphere formation assay:

Cancer cells were seeded with DMEM/F12 media, supplemented with 10% FBS. After 24 hours cells were washed with plain media and conditioned media was added for 48 hrs. After 48

hrs, cells were harvested. Single cell suspension of oral cancer cells was made in serum-free medium (DMEM/F12) supplemented with 20 ng/ml epidermal growth factor (Cat #PHG0311,Sigma), 20 ng/ml bFGF (Cat #PHG0261, Invitrogen), hydrocortisone and B27 (cat #12587010) and mixed with geltrex (Cat #A14132-02). 300 viable cells per well was seeded in ultra-low adherent 96-well plates (Corning, Cat#3474). The growth factors were supplemented on alternate days. 3D spheres were monitored and imaged on the seventh day and quantified using ImageJ software.

Bulk RNA sequencing and pathway analysis:

In brief, CAFs and cancer cells were co-cultured and following co-culture, both the cell types were separated by FACS. RNA extraction was done from a pure population of individual cell types and bulk RNA sequencing was done using Illumina HiSeq2500. Quality assessment was done by using Agilent Bio-analyser 2100 with Agilent nano kit. The library was prepared by TruSeq Stranded Total RNA Library prep kit. The data was processed by the core facility of National Institute of Biomedical Genomics, India. Significant (p value=<0.05) differentially upregulated and downregulated (log2 fold change>= 2) genes were explored for pathway enrichment analysis by Gene Set Enrichment Analysis (GSEA) and Cytoscape software.

Zebrafish xenograft:

Zebrafish were maintained at a 28°C incubator as approved by the NIBMG committee. 48 hrs post fertilized embryos were dechorionated and 100 GFP positive cells were injected in the yolk sac cavity. Post injection, embryos were maintained at 32°C incubator. Fish were monitored and deaths were recorded up to seven days post injection and survival analysis was done using GraphPad prism. Fluorescent imaging was performed using EVOS M7000 and Nikon laser scanning confocal microscopy after anesthetizing using tricaine solution.

Mouse allograft:

Five-week-old C57/BL6 mice were subcutaneously inoculated with $3*10^5$ MOC2 cells exposed to CAF conditioned media. Mice were housed in sterile cages, maintained in a temperature-controlled room and were fed autoclaved food and water. Ten days post cell inoculation, tumors were harvested by sacrificing the mice. Volume of the tumors were measured using ImageJ software and graphs were generated using GraphPad prism. All animal experiments

were done as approved by the animal welfare committee of the IISER Kolkata and NIBMG-Kalyani (IISERK/IAEC/2020/014). The maximal tumour size permitted by the ethics committee was 20 mm in largest diameter measurement (in any direction) and we confirm that the maximal tumour size/burden did not exceed the permitted limit.

Immunofluorescence:

CAFs were plated on poly-D-Lysine and Collagen-I coated 100 mm tissue culture dishes. Cells were fixed with 4% paraformaldehyde at room temperature and washed with ice cold PBS. Permeabilization and blocking was done with 0.2% Tween 20 and 10% goat serum in PBS respectively. Cells were incubated with primary antibodies targeting aSMA (ab7817, 1:300), Ki67 (ab16667, 1:100), Tie2 (A7222,1:100), pTie2 (AF2720, 1:100), BMP4 (ab39973, 1:150) overnight at 4 ^{o}C. Cells were incubated with alexa Fluor conjugated goat anti-mouse and goat anti-rabbit secondary antibodies for one hour at room temperature at a dilution of 1 µg/mL. Nucleus was counter-stained with DAPI (ProLong® Diamond Antifade Mountant, Cat. # P36962 Thermo). Image acquisition was done by EVOS M7000 (ThermoFisher) and Nikon confocal laser scanning microscope (Ti2, Nikon, Japan). Cells were quantified using ImageJ software.

Quantification of total and phosphorylated protein:

For total protein quantification, intensity of each cell was measured in a gray scale format and normalized with the mean area of the cells. The formula used for quantifying total protein was "CTCF = Integrated Density – (Area of selected cell X Mean fluorescence of background readings)". Phospho-Tie2 puncta was quantified using ImageJ software. Images were converted in grayscale and length and width of each bright pTie2-puncta was measured. Puncta which were more than 2µ in length were considered as mature phosphorylation marks, anything below was regarded as immature phosphorylation and discarded from the analysis.

Immunohistochemistry:

Surgically dissected oral tumor tissue samples were obtained from Tata Medical Hospital, Kolkata. These sections were used for IHC analysis of aSMA and Tie2 protein expression.

Tissue block was rehydrated, and antigen retrieval was done in the sodium citrate buffer (pH-6). The samples were then incubated with 3% H2O2 for 10 min. Tissue sections were incubated with monoclonal aSMA (1:50) and polyclonal Tie2 (1:50) antibody overnight at 4°C. Tissue sections were incubated with HRP-conjugated secondary antibody and stained with DAB (Vector, Cat# SK-4105), dehydrated and mounted with DPX (Vector, Cat# H-5000). All the pictures were obtained using the EVOS M7000 microscope (ThermoFisher). Stromal aSMA and Tie2 expressions were scored on a scale of 1 to 4 (10%; 10%–25%; 25%–50%, and 50% positive).

Chromatin Immunoprecipitation (ChIP) pull down and ddPCR:

4.3 million cells of C1 CAF with and without TGF-β were cross-linked using 1% formaldehyde for 10 min at 37°C, and then 0.125 M of glycine was added to quench the remaining. After thorough washing with ice-cold PBS, the cell pellets were resuspended in RIPA, and sonicated using covaris (M200) in glass microtubes (microTUBE AFA Fibre Pre-Slit Snap Cap 6X16mm) to obtain DNA fragments between 200 to 1,000-bp. The lysate was then divided into six equal fractions and 5% of it was retained for Input control. Target proteins were precipitated using antibodies against HDAC2 (ab124974), P300 (ab14984), H3 (ab176842), Lys-27 trimethylated histone H3 (ab6002) and Lys-27 acetylated histone H3 (ab177178) (all purchased from Abcam, MA, USA). Normal IgG was used as negative control. Antibody and lysate were mixed by rotating overnight in 4°C at 13 rpm. Protein A/G-magnetic beads washed with 0.01% PBST followed by ChIP dilution buffer. It was then added to each condition and left them to rotate at 13 rpm for 4 h at 4°C to collect the immune-precipitated complexes. After washing, the beads twice with each buffer i.e. ChIP low salt buffer, high salt buffer, LiCl Buffer and TE Buffer, the DNA was eluted using the elution buffer and kept overnight at 65°C, 650 rpm for de-crosslinking. The following day elutes were treated with proteinase K for 3h at 56°C, 650 rpm. DNA was extracted by the phenol/chloroform method and ethanol precipitation and re-suspended in 50 μl water for CHIP samples and 200 μl for input-control. ddPCR was carried out to check for the presence of histones at C1-signature gene, BMP4 (between region -708bp and -628bp) & ANGPT2 (-360bp & -1600bp) region.

The ChIP pulldown DNA and Input-control DNA was used to perform digital droplet PCR. Evagreen Qx200 ddPCR specific reagents were used. The regions selected were TATA binding and Initiator regions for ANGPT2 while in case of BMP4 no specific TATA binding site could be located hence the region near Initiator was chosen to ensure presence of active and suppressing chromatin marks with relatable expression of genes. The entire ChIP results represented were after normalization using IgG value followed by percentage calculation against H3, the positive control for all the experimental pulldowns.

Single cell RNA sequencing library preparation and sequencing: Co-cultured cells were harvested and made a single cell suspension by incubating with accutase in 37°C for 3-4 minutes followed by filtering through a 70 µm cell strainer. After a brief centrifugation at 300g for 3 minutes, cell pellets were resuspended in the cultured low serum media. Using trypan blue, the cells were counted and checked for viability. The suspension having more than 80% cell viability was diluted to a concentration of approximately 1000 cells/µL. Chromium controller (10X Genomics), chromium 3′ library, Gel bead kit and finally the chromium Single cell 3′ chip kit (10X Genomics) were used according to the manufacturer's instruction to make Single cell gel bead-in emulsion and for sequencing library construction. Agilent high sensitivity chips in TapeStation were used to examine the quality of the libraries and the quantification was done using QuantStudio-7 real time PCR. All the libraries were pooled and sequenced as a paired-end manner in Illumina NovaSeq-6000. Sequence depth was kept at an average of 50,000 raw reads per cell and per sample 200 million paired-end reads were kept as a standard measure. Base call files were converted into FASTQ and GRCh38 reference transcriptome was utilized for the read alignment using CellRanger 4.0.0 (10X Genomics). R package Seurat 4.3.0 was used for the quality control (QC) and the subsequent analysis from the generated gene-barcode matrix from CellRanger.

QC, clustering, and cell type annotation: Cells having less than 10% mitochondrial read and total genes expressing in between 400 and 8000 were taken for further analysis. Genes that were expressed in less than 3 cells were discarded. Seurat function 'LogNormalize' was used to normalize the expression for each cell by considering the feature count of each cell divided

by the total count for that cell multiplied by the default scaling factor set by Seurat i.e., 10000. Variance stabilizing transformation (VST) method was implied to identify the most variable genes i.e., 2000 genes for our case, for principal component analysis (PCA). We constructed graph-based clusters using 15 principal components (PCs) with a resolution of 0.75 and visualized by Uniform Manifold Approximation and Projection (UMAP) using Seurat function 'RunUMAP'. The Seurat object metadata automatically then stores cluster identities and the sample information in seurat@ident and seurat@orig.ident slots, respectively. Probable doublets were removed as described previously (Jerby-Arnon et al., 2018) and again clustering was carried out. For cell type annotation we assigned a module score of established gene sets of epithelial and CAF markers (Puram et al., 2017) for each cell by 'AddModuleScore' function which considers the average expression of the gene set in all the cells from all the clusters. Cell types were segregated by higher or lower scores for the module scores. CAF clusters were separated based on higher CAF gene set module scores and lower or negative for epithelial signature scores. The rest of the clusters were taken into account as epithelial cell clusters. We merged the cell type specific clusters by 'merge' function and normalized, scaling, clustering was done again as described earlier.

Identification of DEGs of clusters and gene set enrichment analysis: DEGs were identified using Seurat function 'FindAllMarkers' with co-parameters such as only.pos=F, and logfc.threshold = 0.25 and the enrichment analysis was performed at cluster level. We used 'Enrichr' for gene set enrichment and gene ontology analysis. KEGG and Molecular Signature Database (MSigDB) for hallmark gene sets, were used as reference databases for all the pathway enrichment analysis. For all the gene set enrichment analysis, we used free publicly available GSEA software (http://www.gseamsigdb.org/gsea/msigdb/human/annotate.jsp). For computing signature scores, gene signatures were taken either from Puram et al., 2017, cell or from MsigDB gene sets. Significant pathways (FDR<0.05) were taken for further analysis.

Pseudotime analysis: Trajectory inference was carried out by monocle3 for the cell type specific merged Seurat objects. SeuratWrappers was used to convert the Seurat object to a

cell data set object by the function ‘as.cell_data_set’ to provide the input object for monocle3. The cluster information and the other data calculated in Seurat metadata was copied in the cell data set object for getting started with trajectory building. Pseudotime value for each cell was then stored internally by learn_graph method and starting root node was provided to assign the start of the trajectory by ordering the cells. Differentially expressed genes along the trajectory were then identified by Morans’s I test method built in monocle3. Co-expressed or co-modulated genes were clustered into modules by find_gene_modules function and cluster wise gene module expression were analyzed.

Gene signature scoring / AUC scoring: Gene signature scores were generated by R tool ‘AUCell’ which uses area under the curve (AUC) measurement to construct the extent to which a critical subset of our input gene signatures were expressed within all the expressed genes in all the cells. A rank for all the genes based on the degree of expression were built for each cell by ‘AUCell_buildRankings’ function and then with ‘AUCell_calcAUC’ function we configured a matrix of scores for the specific gene set on each cell of all the clusters. The median value of AUC scores for all the clusters of CAF/epithelial cell type was used for our analysis.

Single sample gene set enrichment (ssGSEA) and survival analysis: The differentially expressed genes (DEGs) in the treated case were obtained using DESeq2 (Love et al., 2014). The DEGs were shortlisted by adjusted p-value < 0.05 and ranked by their log2-fold change. The top 30 of the upregulated and downregulated genes for the bulk samples were selected as the gene sets for further analysis. All the upregulated genes were considered for the single-cell sample. These gene sets were used to score the TCGA-HNSC samples (The Cancer Genome Atlas Network, 2015) using the GSEApy (Fang et al., 2023) implementation of the single sample gene set enrichment analysis (ssGSEA) algorithm (Barbie et al., 2009). This method evaluates the enrichment of the genes in the gene set compared to the background expression and provides a score quantifying the activity of the genes. Patients were stratified into high-scoring and low-scoring groups based on the median ssGSEA scores, and Kaplan-Meier (KM) analysis was done to compare the 5-year Disease-specific survival (DSS).

Statistical analysis: Statistical analysis was performed using R (v 4.4.0) and Graphpad Prism (v 8.4.3.686) softwares. Data were presented as the mean ± SEM. Two sided paired or unpaired T test was performed using Graphpad Prism and statistical significance was considered as follows: (*p ≤ 0.05, **p ≤ 0.01, ***p ≤ 0.001, ****p ≤ 0.0001; ns, not significant). We obtained 'rstatix' (v0.7.2) package in R for computing p value and statistical difference between groups in single cell RNAseq data. AUC scored groups were compared by Wilcoxon rank sum test, with a Bonferroni method adjusted p value. AdjPValue >0.05 was considered as non-significant. Significance for Kaplan-Meier survival analyses was performed with log-rank test. The details of the statistical tests and significance is mentioned in respective figure legends.

**List of primers used in the study:**		
**Gene**	**Primer sequence (5'-3')-Forward**	**Primer sequence (5'-3')-Reverse**
GAPDH	GGTGGTCTCCTCTGACTTCAACA	GTTGCTGTAGCCAAATTCGTTGT
ACTA2	TGGGTGACGAAGCACAGAGC	CTTCAGGGGCAACACGAAGC
SERPINE1	AGAGCGCTGTCAAGAAGACC	AGTTCTCAGAGGTGCGTTGC
Tie2 (TEK)	TGCGAGATGGATAGGGCTTG	AGGATGGGAAAGGCTGTATC
ANGPT2	TTGAACCAAACAGCGGAGCA	GAGAGGGAGTGTTCCAAGAGC
ANGPT1	GAATTTTTGGGGTGCTTGAA	GAGAGGAGGCCCAGTAGCTT
TGFβ	GGTGTCTCAGTATCCCAGGG	AGACGATCTCTCTCCGACC
BMP4	AGCTAGGTGAGTGTGGCATC	GGCTCGAGATAGCTTGGACG
ID1	TTACTCACGCCTCAAGGA	AGGAACGCATGCCGCC
ALDH1A1	GATGCCGACTTGGACAATGC	TCTTAGCCCGCTCAACACTC
OCT4	GACAGGGGGAGGGGAGGAGCTAG G	CTTCCCTCCAACCAGTTGCCCCAAAC

Nanog	CAGCCCCGATTCTTCCACCAGTCCC	CGGAAGATTCCCAGTCGGGTTCACC
CD44	GAAGAAAGCCAGTGCGTCTC	AAATAATCGGGGCTGCCAGG
CK14	CCCAGTTCTCCTCTGGATCG	GCAGGAGAGGGGATCTTCCA
EYA	AGCTTGTTGCATTCCTGTGG	GGACCCGCTTCTCTGAAAGG
FOXF1	CCAGCGAGTTCATGTTCGAGG	GTTGAAGCCGAGCCCGTTC
RUNX2	GCAAGCAGTATTTACAACAGAGG	ACTGTGCTGAAGAGGCTGTT
ITGB1	CGCGGAACAGCAGGCCCGAG	TGAGCAAACACACAGCAAAC
FN1	CCATCGCAAACCGCTGCCAT	AACACTTCTCAGCTATGGGCTT

**List of CHIP primers used :**		
**Gene**	**Primer sequence (5'-3')-Forward**	**Primer sequence (5'-3')-Reverse**
ANGPT2 PROMOTER_F1	AGCAAGATCTAGGGCTGGGTT	CAGTGAGCGCTGAATAGCTG
ANGPT2 PROMOTER_F2	TATTTTGCCAGCTTAGCACGG	CCGATCTGTTACAGGACCCC
ANGPT2 PROMOTER_F3	GGCTGTCTTTCGGTTAGAGC	GTGAGCGCTGAATAGCTGGT
BMP4 PROMOTER_F	CGCTGTCTTTAGGCCTTGGG	GAACACCTTGGTGATGACCC

**List of antibody used:**		
Antibody	Source	Cat no.
Anti-Tie2 antibody	Abclonal	A7222

Anti-pTie2 antibody	R&D	AF2720
Anti-ACTA2 antibody	Abcam	ab7817
Anti-Ki67 antibody	Abcam	ab16667

**List of recombinant peptide used:**		
Recombinant peptide	Source	Cat no.
TGFβ	Gibco	PHG9214
ANGPT2	Gibco	130-07-20UG
ANGPT1	R&D	923-AN-025/CF

**List of Resource used:**		
Resource	Source	Identifier
MsigDB	(Subramanian et al., 2005)	https://www.gsea-msigdb.org/gsea/msigdb
MAPK	M27565	https://www.gsea-msigdb.org/gsea/msigdb/cards/ REACTOME_MAPK_FAMILY_SIGNALING_CASCADES.html
RTK_RAS PI3K	M47962	https://www.gsea-msigdb.org/gsea/msigdb/human/geneset/KEGG_MEDICUS_REFERENCE_GF_RTK_RAS_PI3K_SIGNALING_PATHWAY.html

Angiopoetin receptor pathway	M92	https://www.gsea-msigdb.org/gsea/msigdb/human/geneset/PID_ANGIOPOIETIN_RECEPTOR_PATHWAY.html
Stemness signature	M1834	https://www.gsea-msigdb.org/gsea/msigdb/human/geneset/BOQUEST_STEM_CELL_UP.html
EMT signature	M5930	https://www.gsea-msigdb.org/gsea/msigdb/human/geneset/HALLMARK_EPITHELIAL_MESENCHYMAL_TRANSITION.html
TGFβ Signature	M42505	https://www.gsea-msigdb.org/gsea/msigdb/human/geneset/FOROUTAN_INTEGRATED_TGFB_EMT_UP
Tie2 signature	M11932	https://www.gsea-msigdb.org/gsea/msigdb/human/geneset/REACTOME_TIE2_SIGNALING
Biorender	Biorender	https://www.biorender.com/
Venn	Venn	https://bioinformatics.psb.ugent.be/webtools/Venn/
ImageJ	ImageJ	https://imagej.net/software/fiji/

Cytoscape	(Shannon et al., 2003)	https://cytoscape.org/
GraphPad Prism	GraphPad Prism	https://www.graphpad.com/features
DESeq2	(Love et al., 2014)	https://genomebiology.biomedcentral.com/articles/ 10.1186/s13059-014-0550-8
GSEApy	(Fang et al., 2023)	https://academic.oup.com/bioinformatics/article/ 39/1/btac757/6847088
ssGSEA	(Barbie et al., 2009)	https://www.nature.com/articles/nature08460
Seurat	(Stuart et al., 2019)	https://satijalab.org/seurat/
Monocle3	(Cao et al., 2019)	https://cole-trapnell-lab.github.io/monocle3/
AUCell	(Aibar et al., 2017)	https://bioconductor.org/packages/release/bioc/html/AUCell.html

Supplementary:1

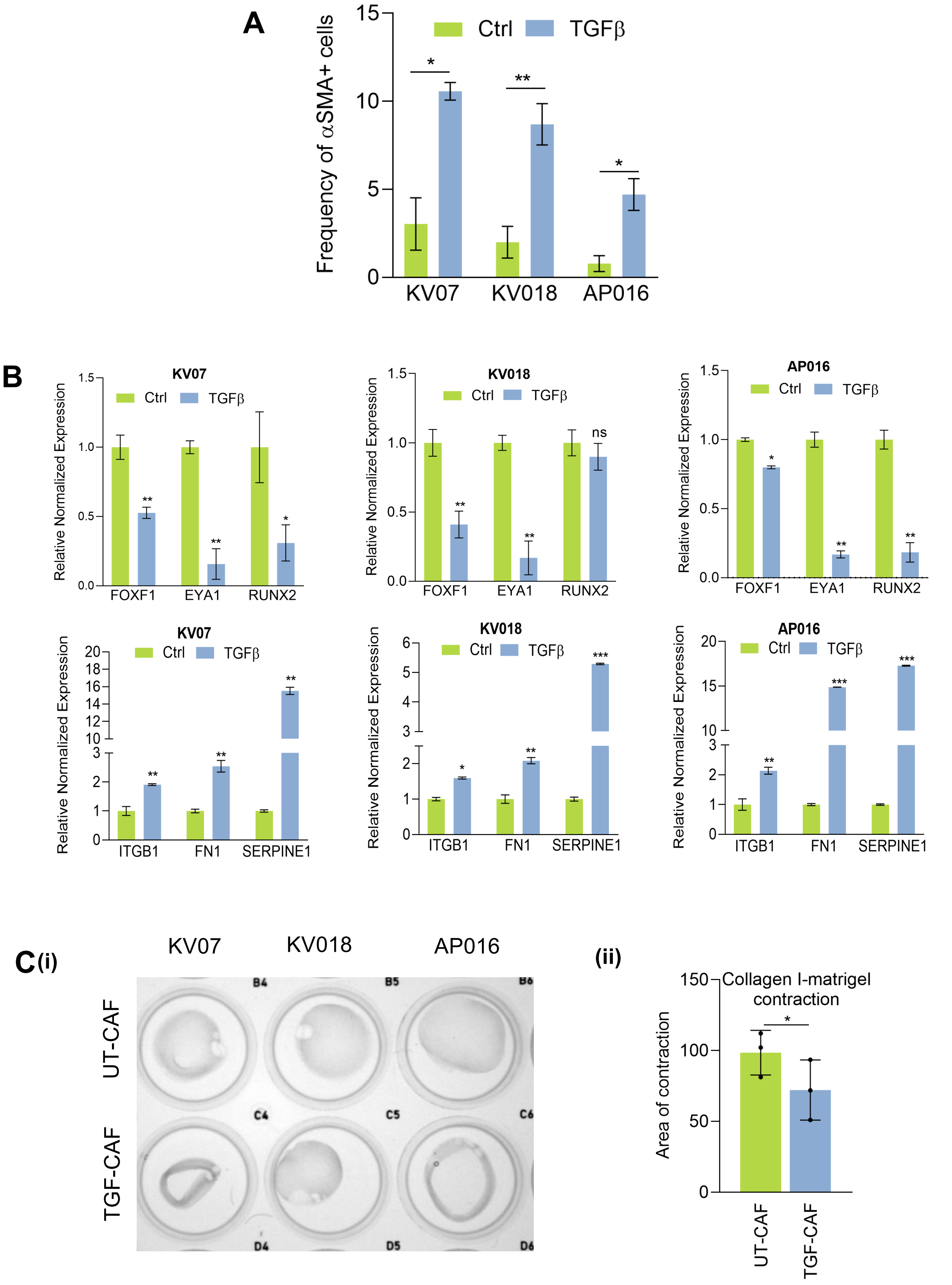

S1: A. Quantification of myofibroblasts frequency following 10 ng/ml TGFβ treatment for 48 hrs. B. qPCR analysis of C1 CAF (*EYA1*, *RUNX2*, *FOXF1*) and C2 CAF (*SERPINE1*, *ITGB1*, *FN1*) markers in CAFs stimulated with 10 ng/ml TGFβ for 48 hrs. C. (i) Image showing 'Collagen I-matrigel' matrix-plug contraction ability of UT-CAF and TGF-CAF. (ii) Result shows Mean± SD of matrix area contraction of three biological repeats (three different CAFs). Paired student T test was calculated. *P<0.05, **P<0.01, ***P<0.001 .

Supplementary:2

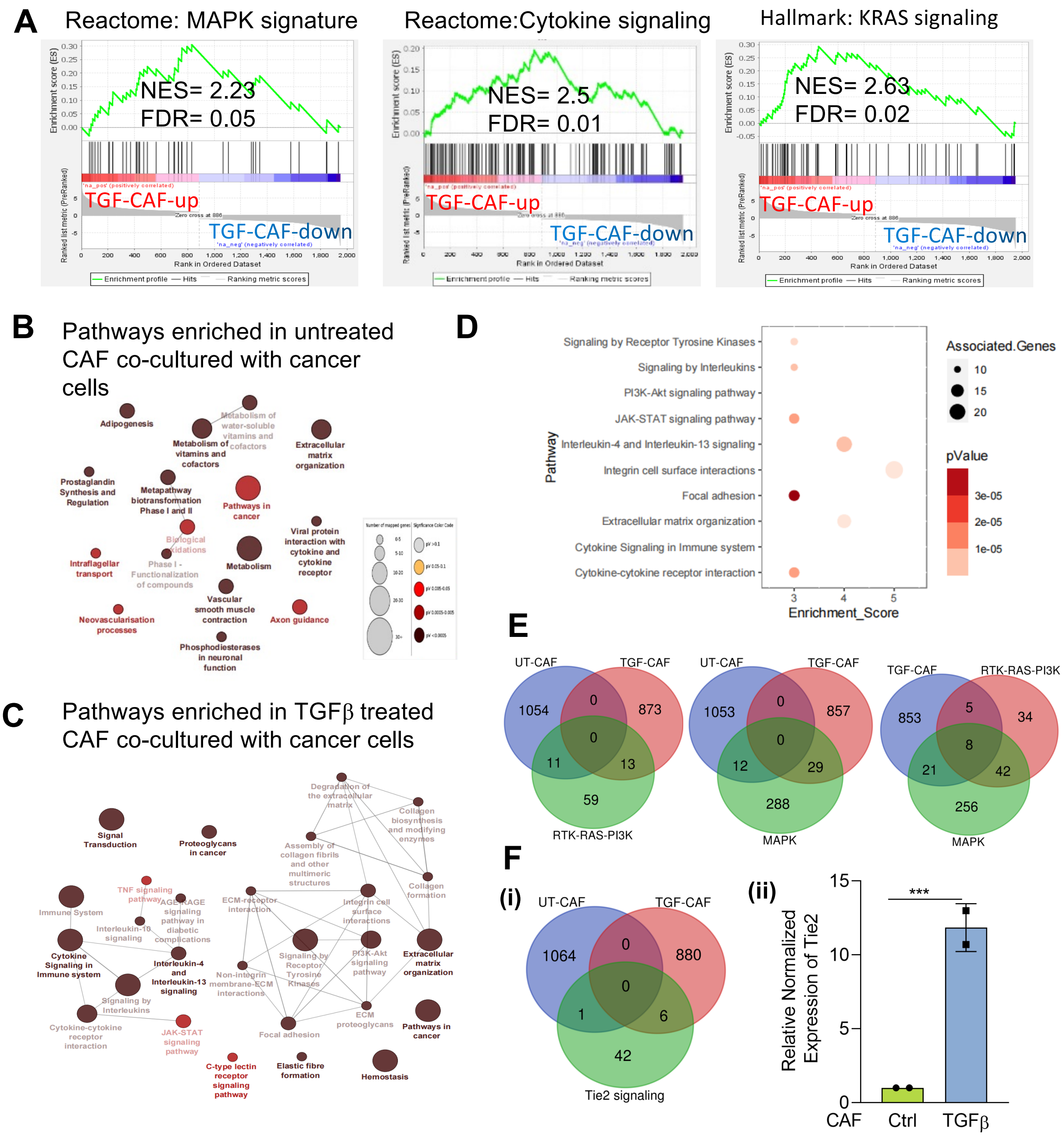

S2: A. Gene set enrichment analysis (GSEA) of CAF stimulated with 10 ng/ml and co-cultured with cancer cells for 4 days. MAP kinase pathway and cytokine-cytokine interaction were positively enriched. Red and blue lines indicated that genes upregulated and downregulated in TGF-CAF co-cultured with cancer cells. Datasets were obtained from MsigDB database. B-C. Cytoscape analysis of positively and negatively enriched networks in TGF-CAF co-cultured with cancer cells. Color coding represents p value and size of nodes represents the number of mapped genes in a particular pathway. D. Pathways shown were found significantly enriched (p value < 0.001) in the TGF-CAF co-cultured with cancer cells. The TGF-CAF signature includes 886 upregulated genes defined with LogFC > 1 and adjusted p value < 0.05 in compared to UT-CAF co-cultured with oral cancer cells. E. Venn diagram showing overlapping gene expression between RTK-RAS-PI3K, MAPK pathway and TGF-CAF co-cultured with cancer cells for 4 days. F. (i) Venn diagram showing overlapping gene expression between Tie2 pathway pathway and TGF-CAF co-cultured with cancer cells for 4 days. (ii) qRT-PCR showing abundance of Tie2 mRNA in TGF- CAF co-cultured with cancer compared to the UT-CAF co-cultured with cancer cells. F. *P<0.05, **P<0.01, ***P<0.001 .

Supplementary: 3

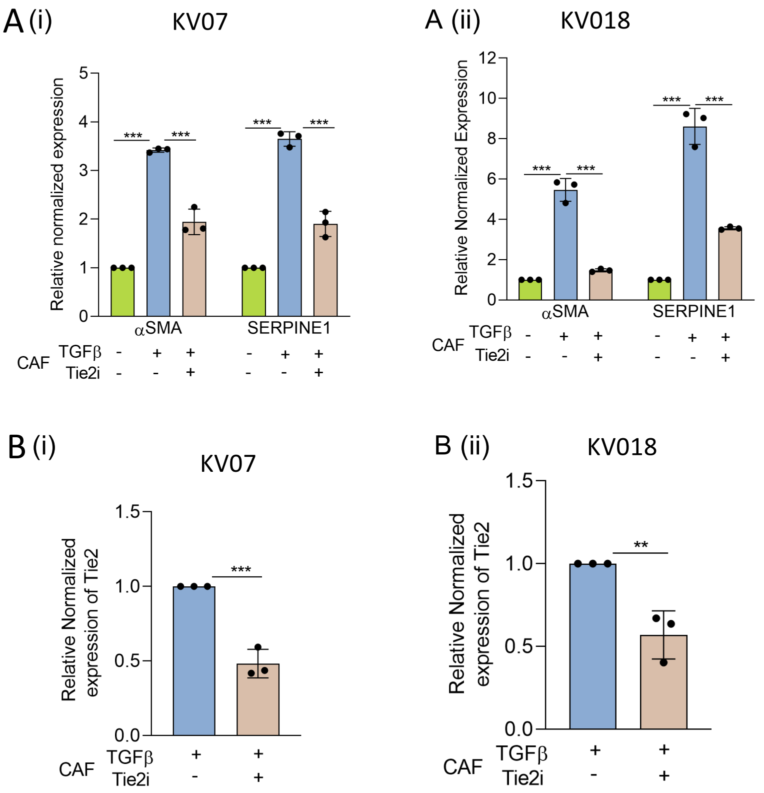

Automated identification of length of pTie2 puncta

Quantification of length of pTie2 puncta

C (i)

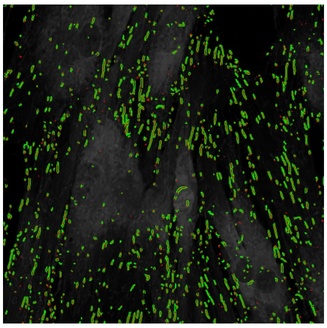

C (ii)

Frame	Contour ID	Length	Mean line width
1	3106	9.905853	0.732067
1	101	13.151321	0.995001
1	3112	0.954326	0.505627
1	3115	0.416027	0.613015
1	3117	2.132074	0.738051
1	105	4.448618	0.66546
1	3119	0.732204	0.517675
1	3122	0.869566	0.73755
1	3125	0.433229	0.494659
1	3128	8.812896	1.124219
1	110	9.865612	1.097046
1	3131	0.532746	0.369801
1	3133	0.876196	0.723449
1	113	7.808697	1.056596
1	114	5.646886	0.663186
1	3135	5.973128	0.910779
1	3137	0.633603	0.43695
1	117	2.900711	0.497118
1	3139	1.36519	0.946723

S3: A(i, ii) qPCR analysis of C2 CAF markers (*αSMA* and *SERPINE1*) following Tie2 inhibition in TGF-CAF of KV07 and KV018 respectively. B(i, ii) Tie2 inhibitor was added for 48 hrs after TGFβ induction (TGF-CAF>>Tie2i) and qPCR for *Tie2* was performed. C(i) Representative images revealing automated quantification of pTie2 puncta. Puncta's with high contrast were measured. (ii) Puncta's length <2 μm were considered immature puncta and discarded from analysis. Puncta's length >2 μm were mature puncta and quantified. Paired student t-test was performed between tested samples; *P<0.05, **P<0.01, ***P<0.001 .

Supplementary: 4

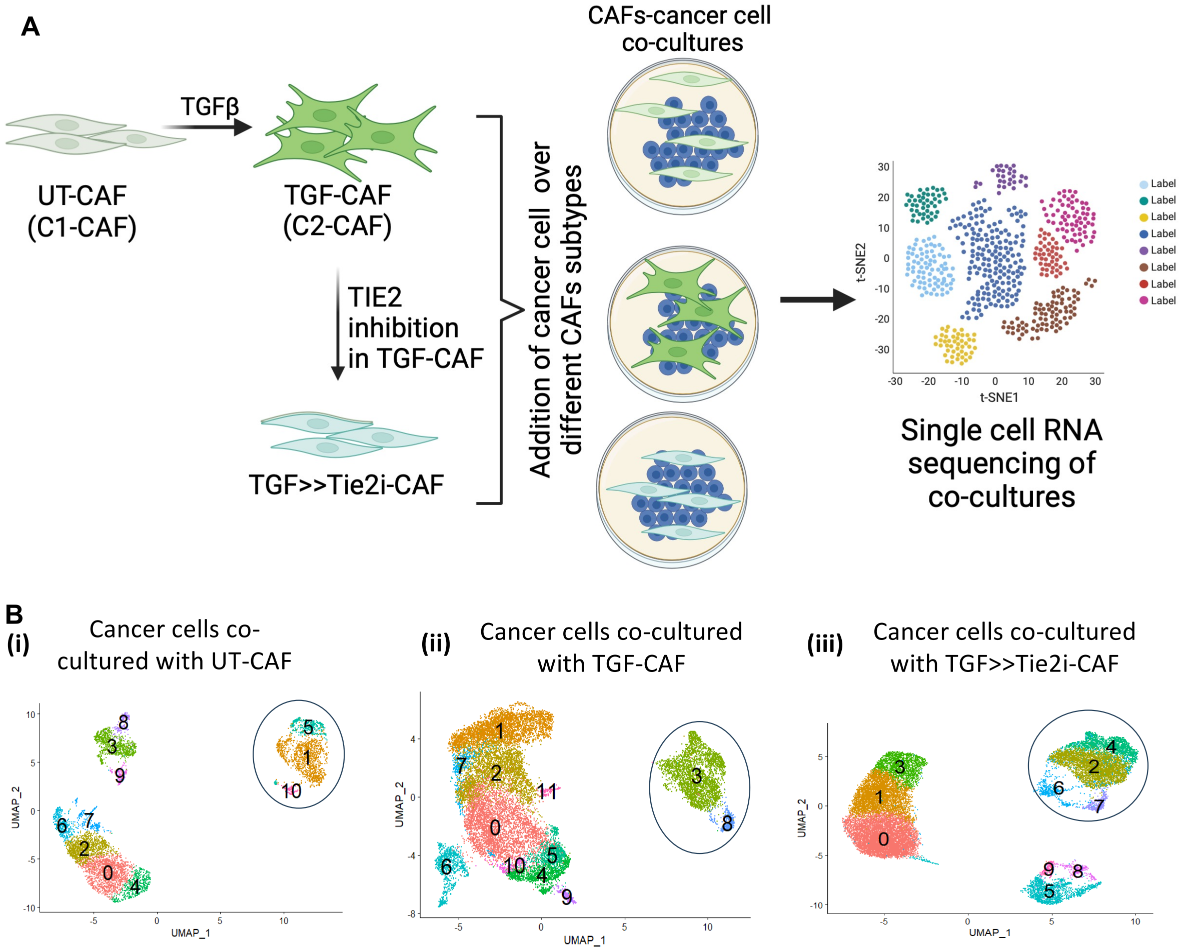

S4: A. Schematic for scRNAseq experiment. B. Clusters of single cells from CAF-cancer cell coculture on UMAP projection. Encircled clusters indicate CAF clusters.

Supplementary: 5

A

Epithelial cell markers	CAF markers
KRT5<br>KRT14<br>KRT17	FAP<br>THY1<br>PDGFRA<br>PDPN

B

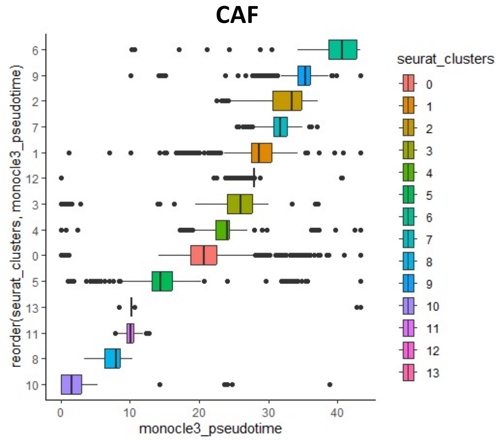

C

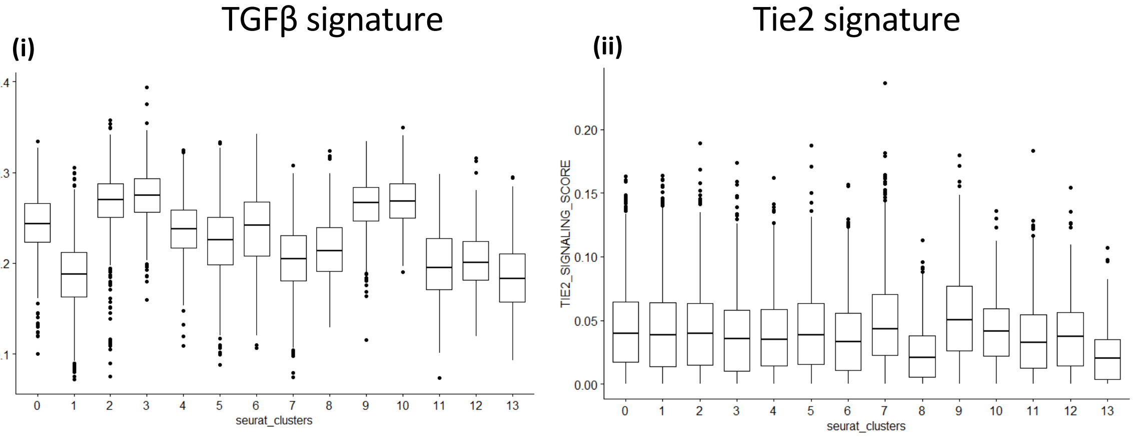

S5: A. List of canonical markers to identify and annotate epithelial and CAF cells in co-cultured scRNA seq clusters. B. Boxplot showing distribution of Monocle3 Pseudotime for each cluster of merged CAF conditions. C. Box plot showing the distribution of TGFβ and Tie2 signaling signature AUC scores across different clusters calculated for each single cell. AUC score measurement was performed by the R tool ‘AUCell’. TGFβ and Tie2 signaling signature was obtained from Molecular Signature Database (MsigDb)

Supplementary:6

Puram patients

A (i)

Patient ID	No. of CAFs	Average Expression		Assignment
		BMP4	ITGA3	
HNSCC13	10	2.266084	0.344597	-
HNSCC6	88	1.95243	0.119921	BMP4 High
HN28	184	1.401452	0.239053	BMP4 High
HNSCC26	27	1.383203	0.077194	BMP4 High
HNSCC28	113	1.302676	0.129819	BMP4 High
HNSCC5	42	1.235971	0.3007	-
HNSCC16	131	0.950274	0.30391	-
HNSCC25	117	0.866	0.432569	-
HN25	37	0.574121	0.475691	-
HNSCC22	54	0.462541	0.62718	ITGA3 High
HNSCC20	13	0.46118	0.285188	-
HNSCC18	50	0.399277	0.272655	-
HNSCC17	43	0.376669	0	-
HN26	37	0.33178	0.636708	ITGA3 High
HNSCC	18	0.311285	0.568692	ITGA3 High
HNSCC8	26	0.264024	0	-
HNSCC24	94	0.126318	0.181314	-
HNSCC10	26	0	0.310183	ITGA3 High
HN23	0	NA	NA	
HNSCC12	1	NA	NA	
HNSCC7	1	NA	NA	
Median		0.518331	0.292944	
Correlation		-0.207503511		

B (i)

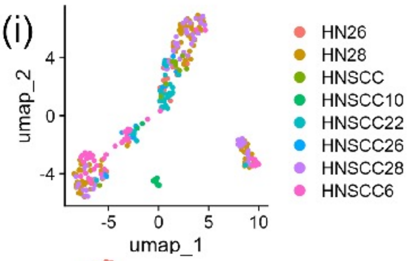

C (i)

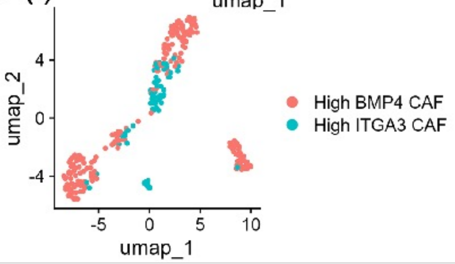

Quah patients

A (ii)

Patient ID	No. of CAFs	Average expression		Assignment
		BMP4	ITGA3	
HN251	677	0.515036	0.211637	-
HN242	89	0.34703	0.071317	BMP4 High
HN263	417	0.231605	0.034463	BMP4 High
HN257	739	0.206165	0.129304	-
HN279	304	0.068377	0.015989	-
HN272	884	0.040865	0.341904	ITGA3 High
HN237	61	0	0.452992	ITGA3 High
Median		0.206165	0.129304	
Correlation		-0.380312131		

B (ii)

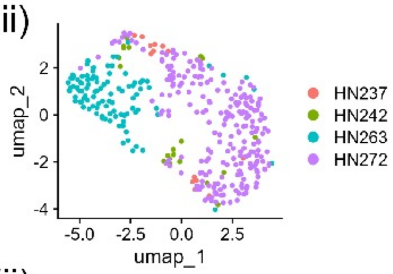

C (ii)

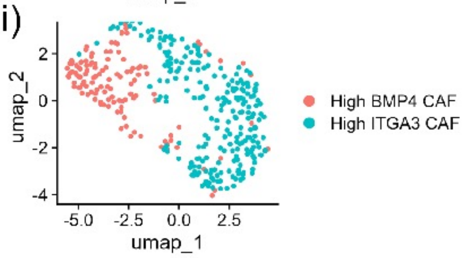

S6: A. (i) Classification of patients from Puram et al. and (ii) Quah et al.as BMP4-High/ ITGA3-High groups with the criterion being above-median expression for one marker and below-median expression for the other. Patients with < 3 identified CAFs and unclassified patients were excluded from further analysis; B. (i - ii) UMAP plots of CAFs from the classified patients, colored based on patient-wise sample-origin, or C. (i-ii) based on assigned grouping.

Supplementary:7

A

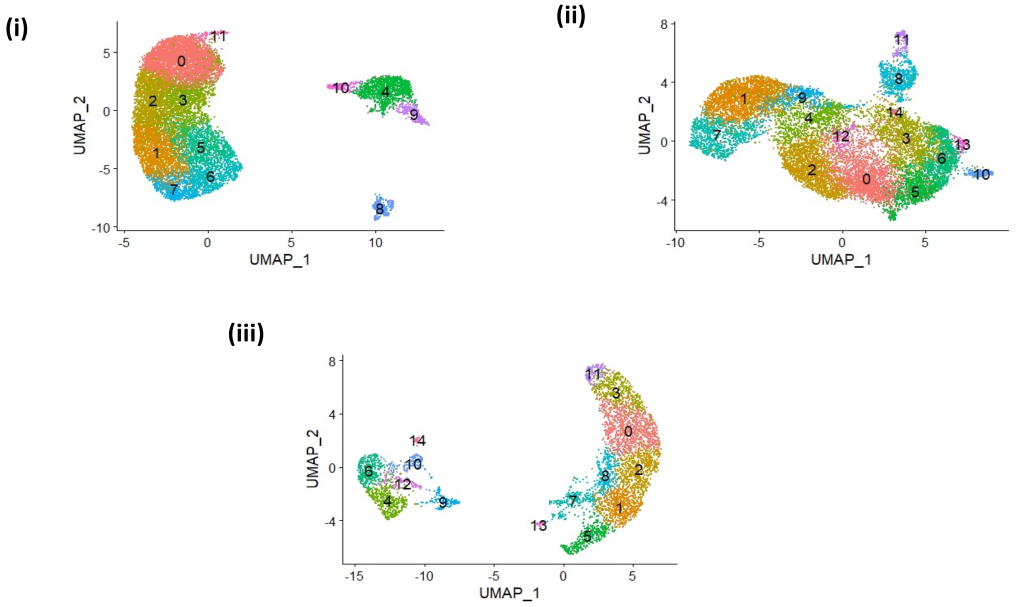

B **Epithelial cells**

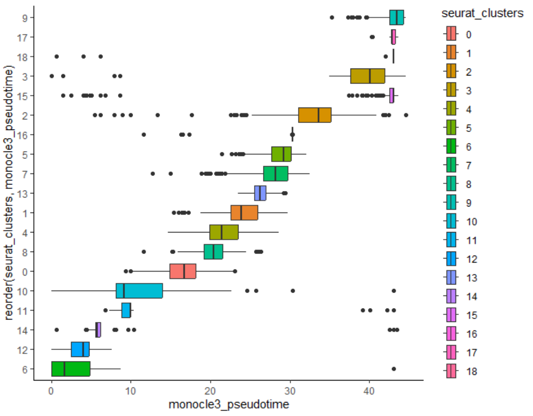

C

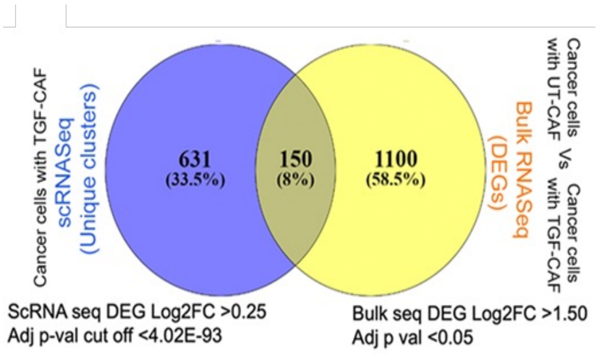

S7: A(i-iii). Epithelial cell cluster subsets separated from the co-cultured UMAP clusters for all 3 conditions. B. Boxplot showing distribution of Monocle3 Pseudotime for each cluster of merged Cancer cell conditions. C. Venn diagram showing overlapping genes between bulk RNAseq DEGs and scRNAseq DEGs of epithelial cells co-cultured with TGF-CAF for 4 days.

Supplmentary 8:

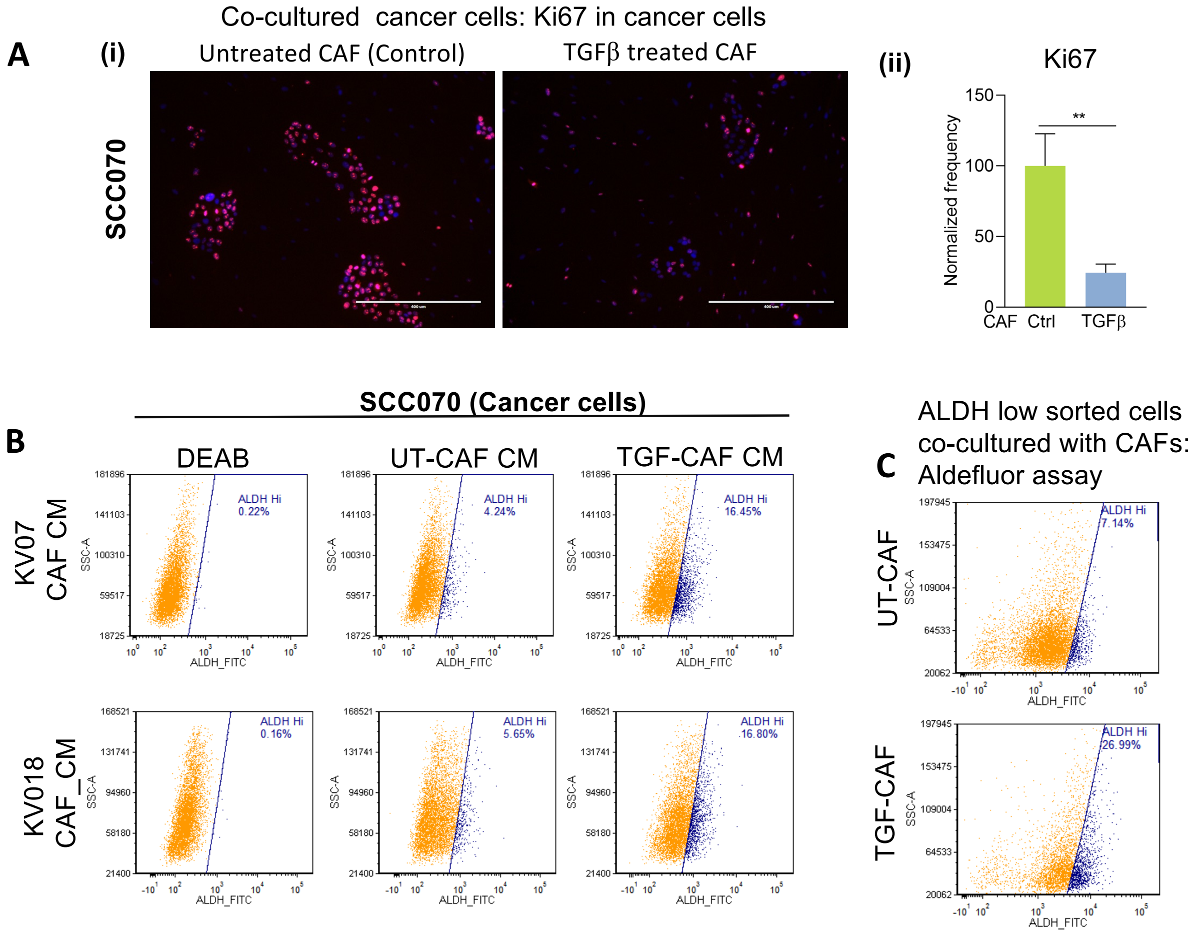

S8: A. (i) Representative IF images of cancer cells were stained for a proliferation marker, Ki67 upon co-culture with UT-CAF and TGF-CAF for 4 days. Nuclei were stained with DAPI. Cancer cells co-cultured with UT-CAF were used as control. (ii) Frequency of Ki67 positive cells were quantified using imageJ software. Paired student t-tests were calculated. B. FACS plot showing the frequency of ALDH positive cancer cells upon exposure to UT-CAF and TGF-CAF conditioned media for 48 hrs. DEAB was used as experimental control and cancer cells exposed to UT-CAF CM was used as biological control. C. FACS plot showing the plasticity of ALDH-low cancer cells when exposed to TGF-CAF converted to ALDH-high cells. ALDH-low cancer cells co-cultured with UT-CAF were used as a control. Paired student t-test was calculated. *P<0.05, **P<0.01, ***P<0.001, ****P<0.0001 .

Supplementary: 9

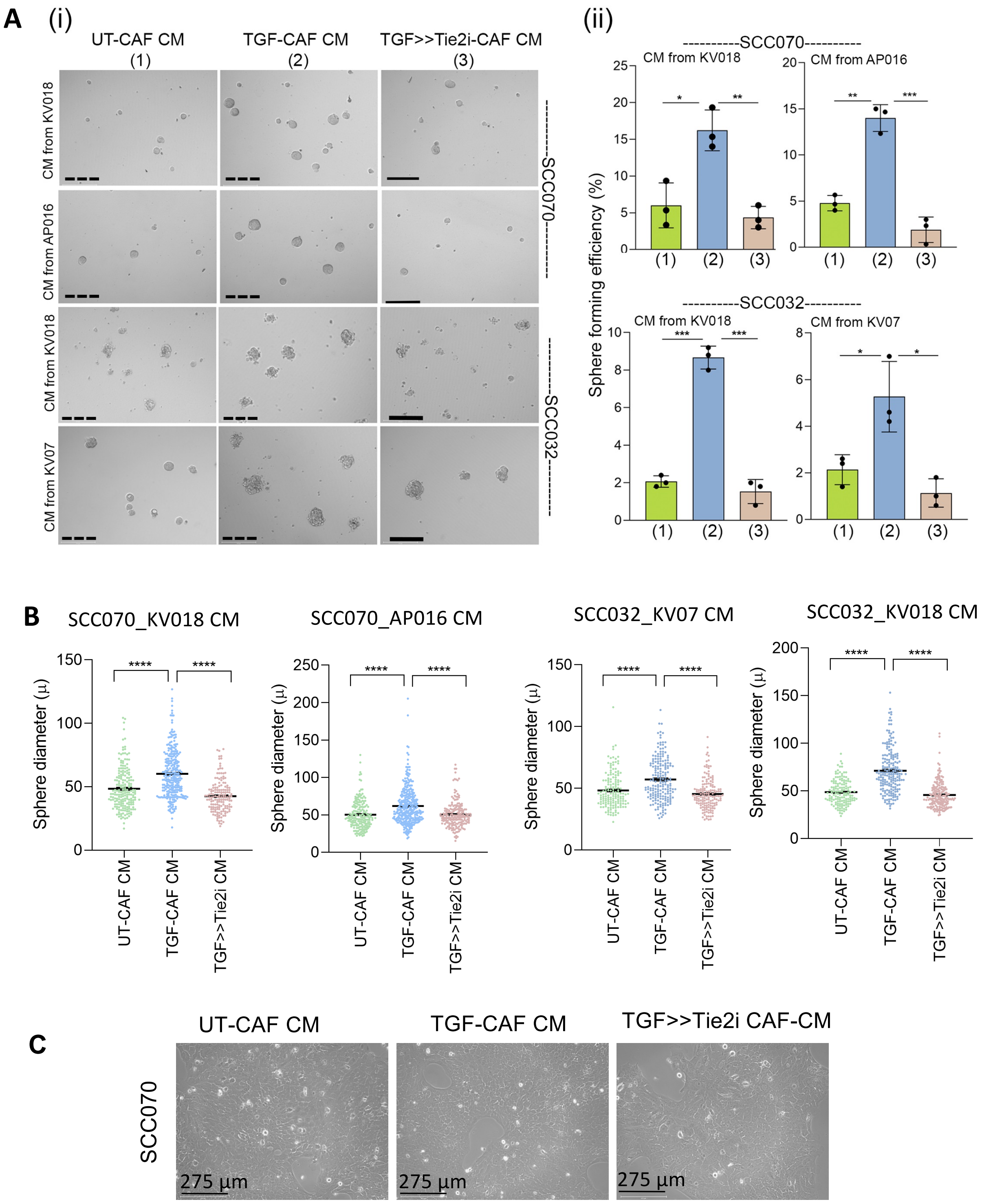

S9: A. (i) Representative images of 3D spheroids of SCC070 cell line exposed to UT-CAF, TGF-CAF and TGF>>Tie2i-CAF conditioned media followed by testing in spheroid formation assay. Spheroids size was quantified using ImageJ. Spheroids of <60 µm diameter were excluded from study. (ii) Sphere forming frequency were calculated for the same. B. Dot plot revealing generated sphere diameter of SCC070 cells exposed to UT-CAF, TGF-CAF and TGF>>Tie2i CAF conditioned media. Cancer cells were plated in complete media for 24 hrs and media was replaced with CAF CM and kept for 48 hrs, harvested and checked for sphere forming ability up to day7. Growth factors were replenished every alternate day. Sphere size was measured and quantified using ImageJ. Statistical difference was found, as calculated by Mann-Whitney test. C. Representative phase contrast images of cancer cells exposed to UT-CAF, TGF-CAF and TGF>>Tie2i CAF conditioned media in monolayer culture. Scale bars, 275 µm *P<0.05, **P<0.01, ***P<0.001, ****P<0.0001 .

Supplementary:10

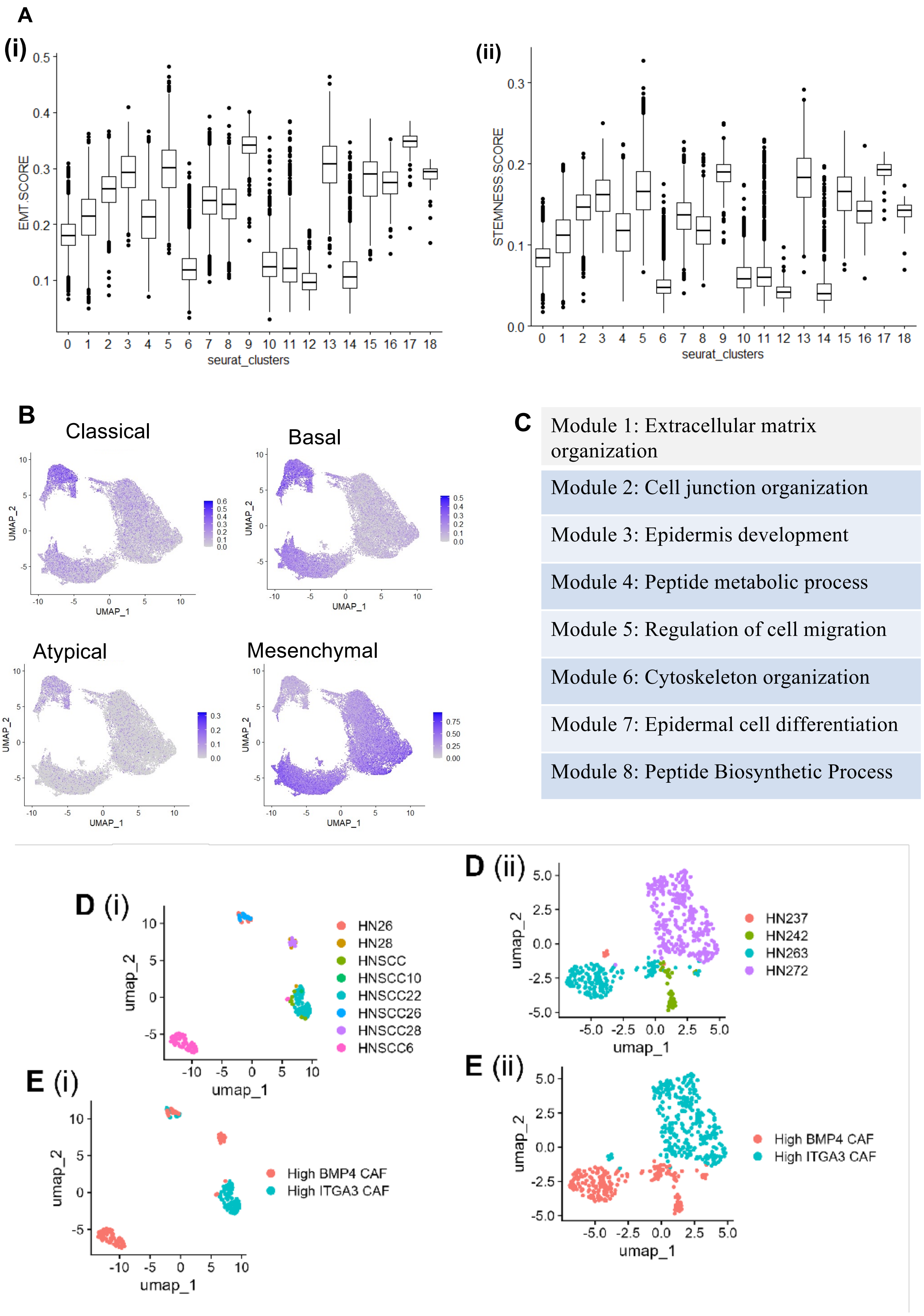

S10: A. (i-ii) Box plot showing the distribution of stemness and EMT (Epithelial to Mesenchymal Transition) signature AUC score across different clusters of merged cancer cell subset from 3 conditions. AUC score measurement was performed by the R tool 'AUCell' . B. Feature plot showing the distribution of classical, basal, atypical and mesenchymal signature AUC score across different clusters of merged cancer cell subset from 3 conditions. AUC score measurement was performed by the R tool 'AUCell'. C. Annotations of co-regulatory gene modules from GO-BP. D. (i - ii) UMAP plots of cancer cells from the classified patients, colored based on patient-wise sample-origin, or E. (i - ii) based on assigned grouping. *P<0.05, **P<0.01, ***P<0.001, ****P<0.0001

Supplementary:11

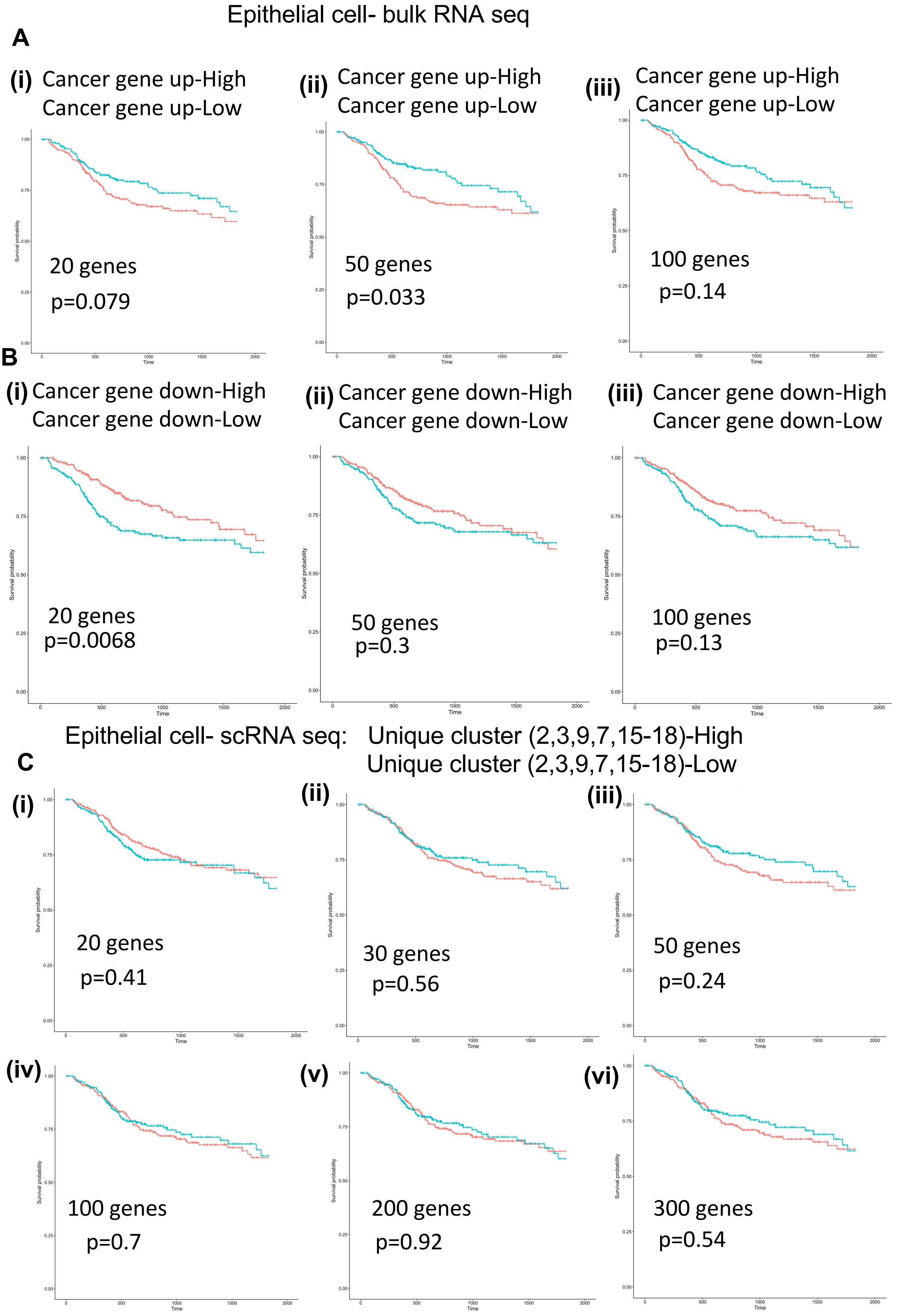

S11: A-B. Kaplan-Meier plot showing survival probability of HNSC patients harbouring gene expression of cancer cells co-cultured with TGF-CAF. Top 20, 50 and 100 upregulated and downregulated genes were taken for single sample gene set enrichment analysis (ssGSEA). C. i-vi. Survival probability of TCGA HNSC patients expressing Top 20,30,50,100,200,300 degs of exclusive cluster (2,3,9,7,15-18) of cancer cells. Red line and blue line indicates upregulation and downregulation of specific gene signatures respectively.
